# Associations between work-privacy conflict and parental relationship satisfaction two years after childbirth: unveiling the moderating role of personality

**DOI:** 10.1186/s12889-026-28783-2

**Published:** 2026-07-30

**Authors:** Sophia Schmidt, Isabel Jaramillo, Judith T. Mack, Victoria Weise, Eva Asselmann, Susan Garthus-Niegel

**Affiliations:** 1 Institute and Policlinic of Occupational and Social Medicine, Faculty of Medicine, TUD Dresden University of Technology, Dresden, 01307 Germany; 2 Faculty of Health, HMU Health and Medical University, Potsdam, Germany; 3 Institute for Systems Medicine, Faculty of Medicine, Medical School Hamburg, Hamburg, Germany; 4 Department of Childhood and Families, Norwegian Institute of Public Health, Oslo, Norway

**Keywords:** DREAM study, Work-privacy conflict, Work-family conflict, Personality, Relationship satisfaction

## Abstract

**Background:**

Previous studies have shown that work-privacy conflict (WPC) can have detrimental effects on mental and physical health, interpersonal relationships, and family functioning. However, these detrimental effects vary widely between individuals. At the same time, there is evidence that the Big Five personality traits interact with how people cope with stress and conflict, so they may partly explain these differences. Thus, this study aims to examine the moderating role of the Big Five personality traits in the association between WPC and relationship satisfaction in mothers and fathers.

**Methods:**

Data from 686 mothers and 702 fathers were derived from a longitudinal cohort study from Eastern Germany. Established questionnaires were used to measure personality at eight weeks, WPC at 14 months, and relationship satisfaction at two years after childbirth. Hierarchical linear regression analyses were performed including relevant confounding variables.

**Results:**

WPC alone did not predict maternal relationship satisfaction, whereas it significantly interacted with neuroticism. Mothers with average and high neuroticism levels showed consistently low relationship satisfaction, irrespective of their perceived WPC. Mothers with low neuroticism levels had low relationship satisfaction when their WPC was high. For fathers, WPC alone significantly negatively predicted relationship satisfaction, yet there was no significant interaction effect with personality.

**Conclusions:**

The present findings indicate a complex interplay between WPC, neuroticism, and relationship satisfaction in mothers, including that average or high neuroticism scores alone are a potential risk factor for relationship satisfaction. Additionally, our results suggest that WPC may have a detrimental effect on relationship satisfaction in fathers, regardless of their personality traits. Based on our results we recommend developing and expanding measures related to work-family benefits to ensure balance between both domains. We also suggest prevention and intervention in the field of positive psychology.

**Supplementary Information:**

The online version contains supplementary material available at 10.1186/s12889-026-28783-2.

## Theoretical background

### Background

Independence, empowerment, or financial necessity – parents’ reasons to work are many and varied. The vast majority of OECD countries reported that many to most of their parents both work [1]. In Germany, both parents were employed in almost 70% of couples with children in 2022 [2]. A trend toward increasing numbers of dual-earner couples with children under the age of three years has been observed for several years [3]. Being employed and taking care of young children goes along with facing potentially conflicting work and family duties and overlapping social roles. This can result in role conflicts, eventually causing burden, exhaustion, and stress [4]. A related consequence of struggling to balance these two important and time-consuming responsibilities can be work-privacy conflict (WPC). WPC, also referred to as work-family conflict, interference of work and family, work-family spillover, or work-life conflict, describes the adverse influence of work-domain experiences on the private domain and vice versa [5–7].

Despite being essentially influenced by work and its characteristics, e.g., job stress or hours spent at work [8], WPC is also determined by private circumstances. Being a parent, for example, can lead to experiencing more WPC compared to being childless [9]. This is consistent with evidence showing that WPC follows a curvilinear trajectory across the lifespan, with elevated levels in mid-adulthood [10]. Therefore, starting a family or having children may increase WPC due to undertaking additional responsibilities in private and family life. The return to work after parental leave is of particular interest, as the family responsibilities that previously received undivided attention now come into conflict with the added demands of the job [11].

Just as WPC has multifaceted antecedent factors, it may also affect multiple essential aspects of life. Meta-analytical evidence shows that WPC may have detrimental effects on work-related outcomes, such as work satisfaction, performance, and commitment [12]. This evidence follows the matching-domain hypothesis, stating that WPC tends to affect outcomes in the domain in which it originates [12, 13]. However, it has been repeatedly shown that WPC also spills over into personal and private domains of life, leading to depression, health problems, stress, and can affect the whole family system by impairing children’s mental health, parent-child-bonding, and satisfaction with one’s own family and relationship [12, 14–19].

### Personality

One of these impactful factors that has been shown to be related not only to WPC, but also to relationship satisfaction, is personality. Including personality may be an approach to explain different perceived levels of WPC even independent of objective work characteristics. Personality plays a major role in how people perceive and deal with stress and challenges in their daily lives [20]. When broken down into the scientifically established Big Five personality traits, i.e., agreeableness, conscientiousness, extraversion, neuroticism, and openness to experience [21], multiple associations have been investigated. Regarding WPC, there is meta-analytical evidence suggesting agreeableness, conscientiousness, and extraversion as being possibly negatively associated with factors of WPC [22]. Conversely, neuroticism has been frequently shown to positively predict WPC [22–24]. Significant findings on openness to experience and WPC are scarce. In contrast, all Big Five personality traits have been linked to various relationship outcomes. Agreeableness and conscientiousness have been both repeatedly shown to positively predict relationship satisfaction [25–27] and related constructs such as social investment in family life [28]. Research on extraversion also indicates positive associations with relationship satisfaction [27, 29]. Furthermore, extraversion is positively associated with related constructs such as marital adjustment [30], relationship quality [31], and support interactions (e.g., giving practical advice or offering help in response to stressful events) among married couples [32]. Additionally, more extravert partners show less jealousy [33]. Contrarily, neuroticism has been shown to be strongly negatively associated with relationship satisfaction [25, 27, 34]. Beyond that, neuroticism has been found to be linked to poorer relationship functioning, which can manifest in maladaptive relationship processes such as poor partner support or an imbalance of power and control in the relationship [35]. In contrast, regarding openness to experience and its link to relationship satisfaction, research and significant findings are scarce. However, it has been found to be positively related to men’s relationship quality [36] and men’s marital adjustment [37], with both constructs being closely related to relationship satisfaction [38].

Although personality is evidently linked to WPC and relationship satisfaction respectively and appears to be an important moderator of associations involving both variables separately [39–41], it has not yet been investigated as a moderator between WPC and relationship satisfaction. This gap will be addressed in this study, as personality may provide an additional perspective on the association between these constructs.

### WPC, personality, and relationship satisfaction in mothers and fathers

Beyond personality, sex is another individual characteristic that is heavily discussed to have an impact on the perception of WPC. Given that research has found that employed German women spend more time on unpaid care and family work than men [42] and that mothers of young children spend less time on paid work than fathers [2], it could be expected that WPC would be perceived differently by women and men. Accordingly, German research found women to be at higher risk of experiencing WPC [5]. However, meta-analytical evidence found no sex differences in the perception of WPC in parents [43], indicating that mothers and fathers are equally exposed to the risk of experiencing WPC with all its consequences. Regarding sex differences in relationship satisfaction, studies also show inconsistent findings [44]. In contrast, personality evidently differs depending on sex [45, 46]. Therefore, the associations between the variables investigated in this study should be investigated separately in mothers and fathers. Given that we aim to explore the interaction of WPC and personality in predicting relationship satisfaction, it is necessary to investigate these effects separately for each group, comparing the results only within their respective samples.

### Aims

This study further contributes to existing research in four ways: Firstly, by integrating personality, this study responds to the call for research on differences in individual characteristics, as they may have an impact on the management of work and family duties [17]. Secondly, by only including parents who recently had a child, this study responds to the call for research using homogeneous samples [8]. Simultaneously, we hence investigate WPC and its possible effects in a life span of particular interest, as the conflicting responsibilities of being employed and a parent of young children may elicit even more challenges in balancing both domains. Especially for mothers, this may be a period in which they recently returned to work after their parental leave, going along with an additional need for readjustment. Thus, investigating this sample separately can provide valuable insights. Additionally, by examining a German sample, this study contributes to the increasing, but still insufficient, research in the European context, as the majority of studies use Northern American samples [5]. Finally, the call for longitudinal research in the context of WPC and its consequences is met [17, 47].

Specifically, the present study aims to fill these gaps by investigating whether individual personality traits moderate the association between one’s own WPC at 14 months after childbirth and one’s own relationship satisfaction at two years after childbirth among working mothers and fathers. Given that WPC, personality, and relationship satisfaction have not yet been investigated together in this context, we aim to lay an important foundation in this field of research by explicitly focusing on intra-individual processes and evaluating whether the effect of WPC on later relationship satisfaction varies as a function of personality. The research question is of specific importance, as relationship satisfaction has been found to influence several dimensions of life. It has not only been shown to affect crucial aspects of one’s own life, such as mental and physical health [48–50], research also indicates that it affects several aspects of family life, such as family functioning [51], child development [52], and children’s academic performance [53]. Therefore, relationship satisfaction is a critical topic that needs to be further explored in terms of influencing factors and antecedents.

The following hypotheses are proposed for mothers and fathers, likewise:

- H_1_: Higher WPC at 14 months after childbirth is linked to lower relationship satisfaction at 24 months after childbirth.
- H_2_: The association between WPC and parental relationship satisfaction varies by personality. Specifically, the association between WPC and relationship satisfaction increases with higher neuroticism but decreases with higher agreeableness, conscientiousness, extraversion, and openness to experience.

### Confounding variables

In addition to the main variables of our study (WPC, personality, and relationship satisfaction), we examined the other variables collected in our data set and checked for potential associations with relationship satisfaction (the outcome) in previous literature. Following variables have been shown to be associated with relationship satisfaction in prior research and were therefore included as confounding variables: education e.g., having an academic degree [54, 55], number of children [56–59], relationship duration [58, 60], perceived social support [61–63], the COVID-19 pandemic [64], employment status two years after childbirth, and expecting another child two years after childbirth [65].

## Methods

### Study design

This study was based on data from the prospective multi-method cohort Dresden Study on Parenting, Work, and Mental Health (DREAM; “DResdner Studie zu Elternschaft, Arbeit und Mentaler Gesundheit”). DREAM aims to longitudinally examine parental work participation, role distribution, stress factors, and how these affect perinatal outcomes and the long-term mental and somatic health of the family [66]. It currently comprises six measurement points: during pregnancy (T1), eight weeks after the anticipated birth date (T2), 14 months (T3), two years (T4), three years (T5), and 4.5 years (T6) after childbirth. Data were collected via print and online surveys consisting of established, validated questionnaires and self-designed items. Participants were recruited from June 2017 to the end of 2020, resulting in a total community sample of *N* = 3,860 expectant parents (*n* = 2,227 birthing mothers, *n* = 16 female partners, and *n* = 1,167 male partners) from Dresden, Germany, and surrounding areas. They were mainly actively recruited at birth information evenings in obstetric clinics and midwife practices, where DREAM study staff delivered presentations. With participants’ consent, T1 questionnaires were distributed on-site, and subsequent questionnaires were sent by mail or email. Additionally, participants were passively recruited, e.g., through recruitment materials displayed in doctors’ offices and midwifery practices. All participants provided written informed consent to participate in this study. For more details on design and procedure, see the study protocol [66]. The study was approved by the Ethics Committee of the Technische Universität Dresden (No. EK 278062015). For the present study, data from T2, T3, and T4 were used. The current study is based on version 10 of the quality-assured data files. At the time of data extraction (March 10, 2023), prospective data collection was complete for T1-T3 and ongoing for T4-T6.

### Sample

**Fig. 1:**
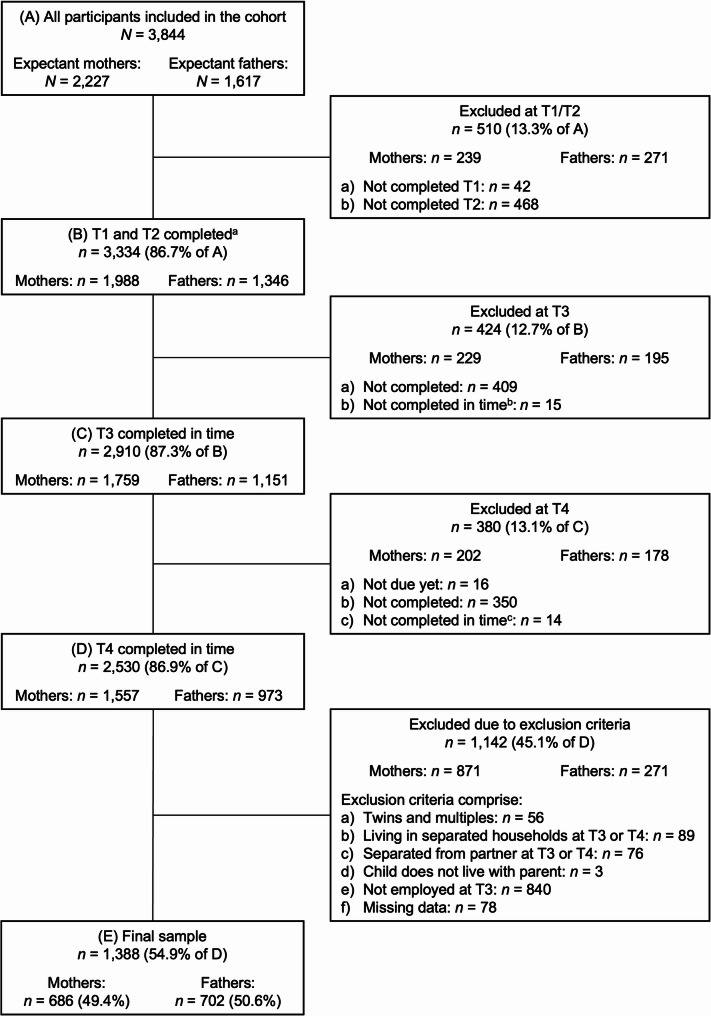
Flowchart of retention rate and exclusion criteria resulting in final sample. Note. T1 = during pregnancy (assessment of sociodemographic variables and the confounder variable academic degree); T2 = around 8 weeks after the anticipated birth (assessment of the moderating variables of the Big Five personality traits); T3 = around 14 months after the actual birth date (assessment of the predictor work-privacy conflict and the confounder variables number of children, relationship duration, and perceived social support); T4 = around 2 years after the actual birth date (assessment of the outcome variable relationship satisfaction and the confounder variables employment status, expecting another child, and COVID-19 pandemic exposure). Data for the current study were extracted on March 10, 2023 (version 10 of the quality-assured data files). Prospective data collection was complete for T1, T2, and T3, and ongoing for T4. ^a^ Three fathers did not complete T1 but completed T2 and were therefore included in the sample because this study does not use T1 data for statistical analyses. ^b^ Not within 12 and 16 months after the actual birth date. ^c^ Not within 22 and 26 months after the actual birth date

At the time of recruitment, we categorized the participants as birthing mother, as non-birthing female partner, or as male partner. Since we did not ask for the participants’ social gender, we only refer to their sex. Female partners of a birthing mother of the index child (*n* = 16) were not included in the current study sample. This is because their inclusion in the subsample of mothers would violate the statistical assumption of independence in the regression analyses given that individuals within a couple mutually influence each other. However, including them in the subsample of (male) partners would undermine our intention to separately examine sex-homogeneous samples. Additionally, participants were excluded if they did not complete all questionnaires in time (see Fig. 1). Furthermore, the following exclusion criteria were applied, and participants were excluded if they were: (1) parents of twins or multiples, as the care of more than one child requires more (mental and material) resources, making it incomparable to parents of singletons; (2) living in separate households at T3 or T4, as the relationship satisfaction of cohabiting couples qualitatively differs from that of separated couples [67]; (3) separated from their partner at T3 or T4, to ensure that the respective partner remained the same for all measurement points of the analyses, which is necessary to test for prospective associations; (4) not living with their child, as this study aims to examine the associations between WPC, personality, and relationship satisfaction in the light of parentship and related responsibilities when taking care of a child on a permanent basis; (5) not employed at T3, as employment is needed to perceive WPC, which was also assessed at T3; (6) participants with missing data in relevant variables (WPC, relationship satisfaction, all of the five personality traits, and exclusion criteria). All in all, most participants were excluded due to the employment criterion (*n* = 692 mothers and *n* = 148 fathers). This resulted in a final sample of *n* = 686 mothers and *n* = 702 fathers. For a detailed overview of the sampling process see Fig. 1.

### Measures

Relationship satisfaction (outcome variable) was assessed at T4 to ensure a prospective design by following WPC assessed at T3. It was operationalized with the German short version of the partnership questionnaire (PFB-K) [68]. The questionnaire contains three subscales (controversial behavior, endearment, and commonality/communication) with three items, respectively. Answers ranged on a 4-point Likert scale from (0) “never/very rare” to (3) “very often”. Out of all nine items, a total sum score was calculated, resulting in a maximum value of 27. Higher scores indicate higher levels of relationship satisfaction. If not more than 20% of item values were missing, they were replaced with the participants’ mean value. The scale had a good internal consistency of Cronbach’s ⍺ = 0.81 for mothers and Cronbach’s ⍺ = 0.80 for fathers.

Work-privacy conflict (predictor variable) was measured at T3, as we aimed to investigate the sensitive period in life after having a child and (returning back to) being employed. T3 was the first wave where we could reasonably assume that a sufficient number of participants had returned to employment, given that full parental allowance in Germany will no longer be paid at 14 months after childbirth. WPC was assessed with the corresponding subscale derived from the German version of the Copenhagen Psychosocial Questionnaire (COPSOQ) [69, 70]. The COPSOQ assesses psychosocial work factors and the WPC subscale specifically investigates how work and its demands spill over to and affect private and family life. The WPC scale consists of seven items measured on a 5-point Likert scale ranging from (1) “to a small extent” to (5) “to a large extent”, which were transformed to fit the range 0—100 accordingly to the guidelines in the manual [69]. The WPC mean score was calculated with higher scores indicating higher WPC. According to the questionnaire manual [69] mean replacement was employed for missing values if the participant responded to at least 50% of the items. The scale had a good internal consistency of Cronbach’s ⍺ = 0.82 for mothers and Cronbach’s ⍺ = 0.81 for fathers.

Personality (moderating variables) was measured at T2 with the German short version of the BFI-S [71, 72]. The questionnaire contains five subscales, each reflecting one personality trait (agreeableness, conscientiousness, extraversion, neuroticism, and openness to experience). Every trait was assessed with three items. Answers ranged on a 7-point Likert scale from (1) “strongly disagree” to (7) “strongly agree”. Out of all three items per subscale, a sum score was computed, resulting in a range of possible values between 3 and 21 for each personality trait. Higher scores indicate higher levels of agreeableness, conscientiousness, extraversion, neuroticism, and openness to experience. No mean replacement was conducted due to the small number of items per subscale. The internal consistency (Cronbach’s alpha) of the subscales for mothers was: agreeableness ⍺ = 0.57, conscientiousness ⍺ = 0.59, extraversion ⍺ = 0.83, neuroticism ⍺ = 0.68, and openness to experience ⍺ = 0.64. For fathers, the Cronbach’s alpha per subscale was: agreeableness ⍺ = 0.47, conscientiousness ⍺ = 0.64, extraversion ⍺ = 0.80, neuroticism ⍺ = 0.69, and openness to experience ⍺ = 0.53. Given that Cronbach’s alpha is usually low in scales with few items [73], we also calculated mean inter-item correlations per trait which ranged from *r* = .32 to *r* = .62 in mothers and from *r* = .24 to *r* = .57 in fathers. The majority of the mean inter-item correlations was within the ideal span between 0.20 and 0.40, suggesting that items are both homogeneous enough but also contain unique variance [74]. For a full overview of all values see additional file 1.

Possible confounder variables were assessed prior to the outcome relationship satisfaction (T4) if both feasible and theoretically plausible in order to account for their effects in a prospective manner. *Academic degree* was (only) assessed at T1 and operationalized dichotomously as to whether participants had a bachelor’s degree (or higher) or not. At T3, we assessed *number of children*, *relationship duration*, and *perceived social support*. Perceived social support was measured with the short version (K-14) of the Social Support Questionnaire (F-SozU) [75]. The scale consists of 14 items with answers ranging on a 5-point Likert scale from (1) “strongly disagree” to (5) “strongly agree”. Mean scores were calculated with higher scores indicating higher levels of perceived social support. If the number of missing item values did not exceed 20%, they were substituted with the participants’ mean value. The scale showed an internal consistency of Cronbach’s ⍺ = 0.92 for mothers and Cronbach’s ⍺ = 0.93 for fathers. Beyond that, theoretical and logical considerations prompted us to also include confounders for T4. Hence, at this wave we assessed employment status, expecting another child, and COVID-19 pandemic exposure. The *employment status* was examined dichotomously as being employed or not being employed. Participants were considered employed if they were working full-time, part-time, marginally employed, or in apprenticeship. With assessing employment status specifically at T4, we aimed to account for potential effects of transitioning to unemployment after T3, which otherwise may not be reflected in the subsequent assessment of relationship satisfaction. Beyond, participants were considered as *expecting another child* if they reported that they or their partner were currently pregnant at T4. Hereby, we aimed to account for possible effects of expecting another child on WPC at T4 by potential employment restrictions in mothers and additional responsibilities for fathers. Finally, it was essential to examine whether the timing of data collection in relation to the COVID-19 pandemic may have had an impact on relationship satisfaction as the outcome variable. Therefore, *COVID-19 pandemic exposure* was measured as follows. Participants were grouped into two categories (before/after vs. during the pandemic) relating to the date of completion at T4, i.e., the date when the outcome variable was assessed. Based on the infection rates and the restrictions in place, the period from March 10th, 2020 to January 15th, 2023 was defined as the pandemic period [76, 77]. Data collection for T4 started in July 2019 and ended in March 2023. Those who completed T4 between March 10th, 2020 and January 15th, 2023 were assigned to the “during pandemic” group, otherwise, they were placed in the “before/after pandemic” group.

### Statistical analyses

All analyses were performed with IBM SPSS Statistics 27 [78] and conducted separately for mothers and fathers. Descriptive analyses were conducted for the outcome, predictor, moderator, confounder, and sociodemographic variables. Attrition analyses were calculated to test whether participants who completed all relevant questionnaires up to T4 (“completers”) and those who dropped out by T3 or T4 or did not complete T3 or T4 (“non-completers”) differ significantly on the outcome, predictor, moderator, confounder, or sociodemographic variables. For the comparison of differences in metric variables, t-tests were conducted, while for dichotomous variables, chi-square tests were performed. Due to the high number of tests, the p-values were adjusted using the Bonferroni-Holm correction. Further difference tests were conducted to identify any sex differences in sociodemographic characteristics and all relevant study variables. The p-values were adjusted again using the Bonferroni-Holm correction. Furthermore, intercorrelations between all study variables were tested: Phi-coefficients were calculated for correlations among dichotomous variables and Pearson correlation coefficients were calculated for correlations involving at least one metric variable. Pearson correlations were computed with 1,000 bootstrap iterations to compensate for lacking normal distributions in variables. If potential confounder variables were statistically significantly correlated with the outcome variable either in mothers or fathers, they were included in the regression analyses for both parents to ensure equal variable sets for each analysis. Univariate outliers up to +/– 3*interquartile range were identified via a box plot. Multivariate outliers were identified using Mahalanobis distance and sensitivity analyses were conducted.

For the main analyses, assumptions for multiple linear regression were checked, and due to lacking homoscedasticity, regression analyses were performed with 2,000 bootstrap iterations. Regression analyses were run to test how parental relationship satisfaction is predicted by WPC, personality traits, and the interaction of both. The regression analyses were structured hierarchically to check how the interactions incrementally predict relationship satisfaction above and beyond WPC and personality traits alone. Therefore, the hierarchical regression analyses were constructed with standardized predictors following these four steps separately for mothers and fathers: Model 1 included relevant confounder variables. In model 2 WPC was added. In model 3 the five personality variables were added. Finally, in model 4 the interaction terms of WPC and the personality variables were added.

After that, significant interaction effects were further examined in exploratory post hoc analyses using the PROCESS macro [79], a regression path analysis modeling tool. The tool was used to calculate simple slopes, to investigate the interaction effect for different levels of the moderator variable. Whenever bootstrapping was performed, bias-corrected and accelerated (BCa) confidence intervals (CI) were calculated, and effects were considered significant at *p* < .05 if the CI did not contain zero. Standardized regression coefficients were interpreted as small (≥ 0.10), medium (≥ 0.30), and large (≥ 0.50), based on guidelines proposed by Cohen [80].

## Results

### Descriptive analyses

The sample characteristics are summarized in Table 1. Notably, more than half of the mothers (61.9%) and fathers (57.6%) had an academic degree, showing a higher level compared to the average population of Dresden [81]. Given that being employed was an inclusion criterion for the present investigation, the numbers of full-time and part-time employment at T3 were relatively high. The distribution between both sexes roughly aligns with data from the German population, which showed that employed parents with children under the age of six reported full-time employment in around 28.5% of mothers and 92% of fathers, while 71.5% of mothers and 6.6% of fathers worked part-time [82]. Furthermore, there was no significant sex difference regarding relationship satisfaction. However, mothers reported significantly less WPC than fathers. Additionally, mothers had higher scores in conscientiousness and neuroticism. Tests for sex differences among confounder variables showed that mothers reported higher levels of perceived social support. At T4, only 86.6% of mothers were still employed and significantly differed from fathers, of which only 97.9% reported employment. For a complete overview of tested sex differences see additional file 2.

**Table 1:** Sample description

Sample characteristics	Mothers (*n* ^a^ = 686)	Fathers (*n* ^a^ = 702)
Outcome variable		
Relationship satisfaction at T4 (*M*, *SD*, Range)	18.34 ± 4.64 (3–27)	18.10 ± 4.25 (4–27)
Predictor variable		
WPC at T3 (*M*, *SD*, Range)	29.10 ± 18.11	33.56 ± 17.70
	(0–96.43)	(0–92.86)
Moderator variables		
Agreeableness at T2 (*M*, *SD*, Range)	16.27 ± 2.78 (4–21)	16.09 ± 2.53 (7–21)
Conscientiousness at T2 (*M*, *SD*, Range)	16.93 ± 2.72 (3–21)	16.08 ± 2.86 (5–21)
Extraversion at T2 (*M*, *SD*, Range)	14.25 ± 3.92 (3–21)	13.93 ± 3.95 (3–21)
Neuroticism at T2 (*M*, *SD*, Range)	11.26 ± 3.61 (3–21)	9.25 ± 3.42 (3–20)
Openness to experience at T2 (*M*, *SD*, Range)	13.74 ± 3.59 (4–21)	14.01 ± 3.33 (5–21)
Confounding variables		
Academic degree at T1 (*n*, %)		
Yes	424 (61.9)	396 (57.6)
No	261 (38.1)	291 (42.4)
Number of children at T3 (*M*, *SD*, Range)	1.20 ± 0.46 (1–4)	1.23 ± 0.53 (1–5)
Employment status ^b^ at T3 (*n*, %)		
Full-time employment	234 (34.1)	563 (80.2)
Part-time employment	429 (62.5)	129 (18.4)
Marginal employment	28 (4.1)	13 (1.9)
Apprenticeship	11 (1.6)	6 (0.9)
Relationship duration in years at T3	8.16 ± 4.00	8.25 ± 3.93
(*M*, *SD*, Range)	(1.91–24.09)	(1.8–22.49)
Perceived social Support at T3 (*M*, *SD*, Range)	4.23 ± 0.61 (2.14–5)	4.07 ± 0.71 (1.07–5)
COVID-19 pandemic exposure at T4 (*n*, %)		
Yes	597 (87.0)	611 (87.5)
No	89 (13.0)	87 (12.5)
Employed at T4 (*n*, %)		
Yes	594 (86.6)	687 (97.9)
No	92 (13.4)	15 (2.1)
Expecting a child at T4 (*n*, %)		
Yes	113 (16.5)	120 (17.1)
No	573 (83.5)	582 (82.9)
Sociodemographic variables		
Country of birth at T1 (*n*, %)		
Germany	662 (96.9)	682 (97.8)
Other	21 (3.1)	15 (2.2)
Age at T2 (*M*, *SD*, Range)	30.62 ± 3.66 (20–42)	32.88 ± 4.75 (22–56)

*WPC* Work-privacy conflict

T1 = during pregnancy; T2 = 8 weeks after the anticipated birth date; T3 = 14 months after the actual birth date; T4 = 2 years after the actual birth date

^a^ *n* slightly varies due to missing data of some participants

^b^ Multiple answers possible

### Attrition analyses

Attrition analyses were performed to compare completers (participants who completed T2, T3, and T4) with non-completers (participants who dropped out and only completed T2 and T3 or T2 and T4) regarding outcome, predictor, moderator, confounder, and sociodemographic variables (see additional file 3 for full results). Analyses showed that in both mothers and fathers, completers were significantly more likely than non-completers to report having an academic degree. In mothers, 61.9% of completers vs. 49.2% of non-completers had an academic degree. In fathers, 57.6% of completers and 41.7% of non-completers reported an academic degree. Furthermore, completers in mothers reported significantly less WPC than non-completers.

### Correlational analyses

Table 2 displays the results of the correlational analyses investigating the associations between all variables. Out of the considered variables for potential confounders, mothers showed statistically significant correlations of relationship satisfaction with academic degree, relationship duration, and perceived social support. Fathers showed statistically significant correlations of relationship satisfaction with the number of children, relationship duration, perceived social support, and expecting another child at T4. The potential impact of the COVID-19 pandemic exposure and the participants’ employment status at T4 were both not significantly linked to relationship satisfaction for either mothers or fathers and were therefore not included in further analyses.

**Table 2:** Pearson correlation matrix including outcome, predictor, moderators, and potential confounders

Variable	1.	2.	3.	4.	5.	6.	7.	8.	9.	10.	11.	12.	13.	14.
1. Relationship satisfaction (T4)	–	− 0.14** [-0.23, -0.06]	0.15** [0.08, 0.22]	0.12** [0.04, 0.19]	0.08 [0.00, 0.16]	− 0.15** [-0.22, -0.07]	. 09* [0.01, 0.17]	0.06 [-0.02, 0.14]	− 0.14** [-0.24, -0.05]	− 0.11** [-0.18, -0.04]	0.35** [0.28, 0.42]	0.03 [-0.05, 0.12]	− 0.06 [-0.13, 0.03]	0.14** [0.07, 0.21]
2. WPC (T3)	-0.10* [-0.17, -0.03]	–	− 0.14** [-0.23, -0.07]	− 0.07 [-0.16, 0.02]	0.01 [-0.07, 0.09]	. 18** [0.10, 0.27]	0.10* [0.03, 0.19]	0.10** [0.03, 0.18]	0.09* [0.02, 0.17]	0.05 [-0.04, 0.13]	− 0.12** [-0.21, -0.05]	− 0.03 [-0.11, 0.04]	0.00 [-0.08, 0.09]	0.02 [-0.06, 0.09]
3. Agree-ableness (T2)	0.17** [0.09, 0.25]	− 0.08* [-0.16, -0.00]	–	0.10* [0.02, 0.16]	− 0.02 [-0.10, 0.06]	− 0.16** [-0.23, -0.09]	0.10* [0.01, 0.17]	− 0.03 [-0.12, 0.05]	− 0.02 [-0.08, 0.03]	− 0.06 [-0.14, 0.03]	0.20** [0.12, 0.28]	0.04 [-0.05, 0.13]	− 0.02 [-0.09, 0.05]	0.01 [-0.07, 0.09]
4. Conscien-tiousness (T2)	0.10** [0.02, 0.18]	− 0.03 [-0.11, 0.05]	0.19** [0.09, 0.28]	–	0.18** [0.10, 0.26]	− 0.19** [-0.26, -0.10]	0.10* [0.02, 0.17]	− 0.06 [-0.13, 0.03]	0.05 [-0.03, 0.14]	0.04 [-0.04, 0.12]	0.06 [-0.01, 0.13]	− 0.02 [-0.10, 0.07]	0.05 [-0.05, 0.16]	− 0.03 [-0.12, 0.04]
5. Extraversion (T2)	0.09* [0.02, 0.16]	− 0.03 [-0.11, 0.04]	0.05 [-0.04, 0.13]	0.13** [0.05, 0.22]	–	− 0.15** [-0.23, -0.06]	0.23** [0.15, 0.30]	− 0.09* [-0.17, -0.01]	0.01 [-0.06, 0.08]	− 0.03 [-0.11, 0.05]	0.33** [0.25, 0.40]	0.02 [-0.06, 0.10]	0.04 [-0.02, 0.11]	− 0.01 [-0.09, 0.06]
6. Neuroticism (T2)	-0.18** [-0.25, -0.11]	0.15** [0.07, 0.24]	− 0.16** [-0.23, -0.08]	− 0.17** [-0.24, -0.09]	− 0.26** [-0.34, -0.18]	–	− 0.01 [-0.10, 0.07]	0.07 [-0.01, 0.14]	0.01 [-0.07, 0.09]	− 0.01 [-0.09, 0.07]	− 0.21** [-0.29, -0.14]	− 0.03 [-0.11, 0.04]	0.04 [-0.02, 0.11]	− 0.02 [-0.10, 0.06]
7. Openness to experience (T2)	0.04 [-0.02, 0.12]	0.10* [0.02, 0.18]	0.11** [0.03, 0.20]	0.04 [-0.04, 0.12]	0.26** [0.18, 0.33]	− 0.06 [-0.14, 0.03]	–	0.03 [-0.04, 0.11]	0.05 [-0.03, 0.13]	− 0.07 [-0.15, 0.01]	0.11** [0.04, 0.18]	0.02 [-0.05, 0.09]	− 0.05 [-0.11, 0.01]	0.01 [-0.07, 0.10]
8. Academic degree (T1)	-0.10** [-0.18, -0.03]	0.19** [0.12, 0.25]	− 0.03 [-0.11, 0.05]	− 0.03 [-0.11, 0.04]	− 0.07 [-0.15, 0.01]	0.14** [0.06, 0.21]	0.04 [-0.04, 0.12]	–	− 0.04 [-0.12, 0.04]	0.11** [0.03, 0.19]	0.07 [-0.02, 0.14]	− 0.10* [-0.17, -0.03]	− 0.01 [-0.09, 0.08]	0.14** [0.06, 0.21]
9. Number of children (T3)	− 0.05 [-0.13, 0.03]	0.0 [-0.01, 0.15]	− 0.05 [-0.13, 0.05]	0.02 [-0.06, 0.10]	0.01 [-0.07, 0.08]	− 0.01 [-0.09, 0.07]	− 0.05 [-0.12, 0.02]	− 0.04 [-0.12, 0.04]	–	0.09* [0.02, 0.16]	− 0.16** [-0.24, -0.07]	0.01 [-0.07, 0.08]	− 0.03 [-0.16, 0.07]	− 0.13** [-0.18, -0.06]
10. Relationship duration (T3)	− 0.09* [-0.16, -0.03]	0.04 [-0.03, 0.12]	− 0.04 [-0.12, 0.04]	− 0.03 [-0.11, 0.05]	− 0.04 [-0.11, 0.04]	− 0.02 [-0.09, 0.06]	0.04 [-0.04, 0.12]	0.09* [0.01, 0.17]	0.24** [0.16, 0.33]	–	− 0.06 [-0.14, 0.01]	− 0.01 [-0.09, 0.07]	− 0.03 [-0.12, 0.05]	− 0.10* [-0.17, -0.03]
11. Perceived social support (T3)	0.25** [0.18, 0.31]	− 0.17** [-0.25, -0.09]	0.07 [0.00, 0.15]	0.26** [0.18, 0.33]	0.16** [0.09, 0.24]	0.11** [0.02, 0.19]	− 0.22** [-0.29, -0.15]	0.02 [-0.05, 0.10]	− 0.08 [-0.16, 0.01]	− 0.03 [-0.11, 0.05]	–	0.01 [-0.07, 0.09]	− 0.05 [-0.11, 0.01]	0.06 [-0.02, 0.13]
12. COVID-19 pandemic exposure (T4)	0.04 [-0.04, 0.11]	− 0.05 [-0.13, 0.04]	− 0.01 [-0.08, 0.07]	0.06 [-0.02, 0.13]	0.02 [-0.06, 0.10]	0.07 [0.00, 0.14]	− 0.01 [-0.09, 0.07]	0.00 [-0.07, 0.08]	− 0.01 [-0.08, 0.06]	− 0.03 [-0.11, 0.05]	0.01 [-0.08, 0.09]	–	− 0.02 [-0.06, 0.04]	− 0.07 [-0.16, 0.01]
13. Employment status (T4)	− 0.00 [-0.08, 0.07]	0.01 [-0.06, 0.09]	− 0.00 [-0.07, 0.08]	− 0.02 [-0.10, 0.06]	− 0.02 [-0.10, 0.07]	− 0.06 [-0.14, 0.03]	0.07 [-0.01, 0.15]	− 0.07 [-0.15, 0.01]	0.11** [0.06, 0.16]	0.10* [0.03, 0.16]	0.03 [-0.05, 0.12]	− 0.07 [-0.12, -0.00]	–	− 0.02 [-0.12, 0.06]
14. Expecting another child (T4)	0.05 [-0.02, 0.11]	0.05 [-0.02, 0.12]	− 0.03 [-0.10, 0.04]	0.02 [-0.05, 0.09]	0.05 [-0.02, 0.11]	0.01 [-0.07, 0.09]	0.01 [-0.07, 0.08]	0.07 [-0.00, 0.14]	− 0.18** [-0.21, -0.15]	− 0.02 [-0.08, 0.05]	− 0.03 [-0.10, 0.04]	− 0.05 [-0.14, 0.03]	− 0.13** [-0.22, -0.05]	–

The correlations for mothers (*n* = 686) are shown below the diagonal. Correlations for fathers (*n* = 702) are shown above the diagonal

*WPC* Work-privacy conflict

*N* varies slightly for each correlation coefficient due to missing data of some participants

Correlations were computed with bootstrapping. Values in brackets show bias-corrected and accelerated confidence intervals with α = 0.05, 95% percentile, based on 1,000 bootstrap samples

T2 = 8 weeks after the anticipated birth; T3 = 14 months after the actual birth date; T4 = 2 years after the actual birth date

* *p* < .05. **p < .01

### Regression and moderation analyses

Using Mahalanobis distance, multivariate outliers were identified to examine which participants systematically differed from the rest of the subsample across all variables. This procedure identified four mothers and three fathers. Sensitivity analyses comparing regression results with outliers vs. without outliers did not show differences in the statistical significance of effects or their sizes (see supplement). Accordingly, the results reported here pertain to the analyses including outliers. Tables 3 and 4 show the final model 4 regression results for mothers and fathers. For an overview of the corresponding complete hierarchical regression results see additional files 4 and 5.

**Table 3:** Multiple linear regression of WPC, personality, and their interaction on relationship satisfaction in mothers ^a^

Variable	*B*	*SE*	β	BCA 95% CI	*p*	*R* ^2^	Adj. *R*^2^	*F* for Δ*R*^2^
Model 4						0.12	0.10	1.35
Constant	12.96	1.49		[10.40, 15.52]	< 0.001			
Academic degree	-0.90	0.37	**− 0.09**	[-1.61, -0.16]	0.020			
Number of children	-0.17	0.39	− 0.02	[-1.00, 0.56]	0.666			
Relationship duration	0.00	0.00	− 0.06	[0.00, 0.00]	0.125			
Social support	1.53	0.31	**0.20**	[0.95, 2.11]	< 0.001			
Expecting another child	0.73	0.48	0.06	[-0.13, 1.65]	0.097			
WPC ^b^	-0.01	0.01	− 0.03	[-0.03, 0.01]	0.469			
Agreeableness ^b^	0.17	0.07	**0.10**	[0.04, 0.30]	0.018			
Conscientiousness ^b^	0.07	0.07	0.04	[-0.07, 0.22]	0.303			
Extraversion ^b^	-0.01	0.05	− 0.01	[-0.11, 0.09]	0.793			
Neuroticism ^b^	-0.14	0.05	**− 0.11**	[-0.24, -0.03]	0.010			
Openness to experience ^b^	0.03	0.05	0.02	[-0.08, 0.13]	0.595			
WPC x Agreeableness	0.00	0.00	0.01	[-0.01, 0.01]	0.721			
WPC x Conscientiousness	-0.00	0.00	− 0.01	[-0.01, 0.01]	0.754			
WPC x Extraversion	-0.00	0.00	− 0.02	[-0.01, 0.00]	0.583			
WPC x Neuroticism	0.01	0.00	**0.09**	[0.00, 0.01]	0.025			
WPC x Openness to experience	0.00	0.00	0.01	[-0.00, 0.01]	0.838			

*WPC* Work-privacy conflict, *SE* Standard error for unstandardized beta based on 95% bias-corrected and accelerated bootstrap confidence interval (2,000 iterations), *ß* Standardized beta coefficient, *Adj. R*^2^ Adjusted coefficient of determination

Significant standardized beta coefficients are marked in bold

*n* = 651. The values depicted in the table refer to Model 4 (complete model of the respective hierarchical regressions)

^a^ Controlled for confounders, including multivariate outliers

^b^ Mean-centered

**Table 4:** Multiple linear regression of WPC, personality, and their interaction on relationship satisfaction in fathers ^a^

Variable	*B*	*SE*	β	BCA 95% CI	*p*	*R* ^2^	Adj. *R*^2^	*F* for Δ*R*^2^
Model 4						0.18	0.16	1.06
Constant	11.75	1.19		[9.28, 13.94]	< 0.001			
Academic degree	0.37	0.33	0.04	[-0.34, 1.07]	0.279			
Number of children	-0.60	0.32	− 0.07	[-1.30, 0.17]	0.106			
Relationship duration	0.00	0.00	− 0.07	[0.00, 0.00]	0.072			
Social support	1.76	0.25	**0.29**	[1.23, 2.39]	< 0.001			
Expecting another child	1.20	0.43	**0.11**	[0.42, 1.97]	0.003			
WPC ^b^	-0.02	0.01	**− 0.09**	[-0.04, -0.00]	0.018			
Agreeableness ^b^	0.10	0.07	0.06	[-0.03, 0.22]	0.141			
Conscientiousness ^b^	0.13	0.06	**0.09**	[0.02, 0.24]	0.020			
Extraversion ^b^	-0.06	0.05	− 0.05	[-0.14, 0.02]	0.222			
Neuroticism ^b^	-0.07	0.05	− 0.05	[-0.16, 0.02]	0.189			
Openness to experience ^b^	0.08	0.05	0.06	[-0.02, 0.19]	0.129			
WPC x Agreeableness	-0.01	0.00	− 0.06	[-0.01, 0.00]	0.147			
WPC x Conscientiousness	0.00	0.00	0.02	[-0.00, 0.01]	0.567			
WPC x Extraversion	-0.00	0.00	− 0.03	[-0.01, 0.00]	0.462			
WPC x Neuroticism	0.00	0.00	0.03	[-0.00, 0.01]	0.459			
WPC x Openness to experience	-0.00	0.00	− 0.03	[-0.01, 0.00]	0.487			

*WPC* Work-privacy conflict, *SE* Standard error for unstandardized beta based on 95% bias-corrected and accelerated bootstrap confidence interval (2,000 iterations), *ß* Standardized beta coefficient, *Adj. R*^2^ Adjusted coefficient of determination

Significant standardized beta coefficients are marked in bold

*n* = 632. The values depicted in the table refer to Model 4 (complete model of the respective hierarchical regressions)

^a^ Controlled for confounders, including multivariate outliers

^b^ Mean-centered

Hierarchical regression analyses were conducted to examine the incremental predictive value of WPC, the respective personality variable, and their interaction. Model 1, including only the confounding variables academic degree, number of children, relationship duration, perceived social support, and expecting a child at T4 explained 8% of the variance in relationship satisfaction in mothers and 15% in fathers. Regarding confounders, higher academic degree was related to lower relationship satisfaction in mothers, whereas higher perceived social support predicted higher relationship satisfaction in mothers and fathers. Furthermore, expecting another child at T4 predicted higher relationship satisfaction in fathers. Adding WPC in model 2 did not significantly improve *R*^2^ in mothers, but increased *R*^2^ in fathers. In model 3, all personality variables were included, resulting in significant changes of explained variance in mothers and fathers. Lastly, in model 4 the five interaction terms of WPC with each personality variable were added, not significantly improving the explained variances in mothers and fathers.

After having added all personality variables and interaction terms, WPC did not significantly predict mothers’ relationship satisfaction in model 4. In contrast, in fathers, higher WPC predicted lower relationship satisfaction in model 4. In addition, higher agreeableness and lower neuroticism in mothers as well as higher conscientiousness in fathers predicted higher relationship satisfaction. There were no statistically significant interactions between WPC and personality, except that WPC and neuroticism interacted in predicting relationship satisfaction in mothers. All in all, model 4 accounted for 12% variance in relationship satisfaction in mothers and 18% in fathers.

### Further analyses

**Fig. 2:**
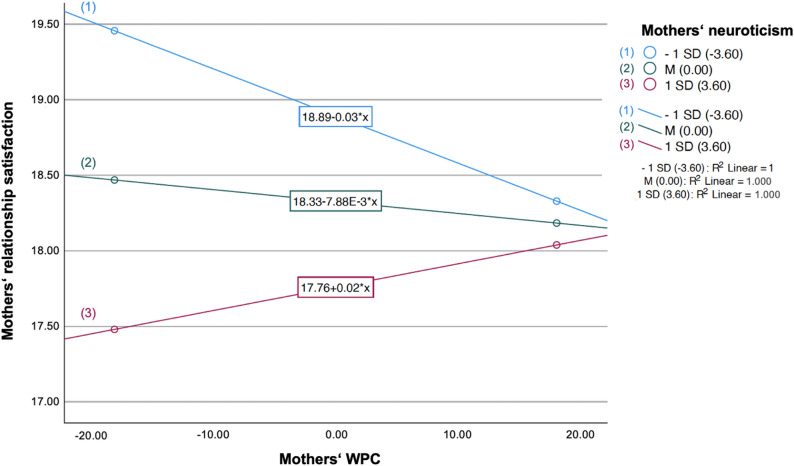
Simple slopes of the interaction of WPC and neuroticism predicting relationship satisfaction in mothers. Note. *n* = 659; WPC = Work-privacy conflict. Calculated unstandardized simple slopes were (1) for neuroticism scores 1 *SD* below the mean: B = -0.03 (BCa 95% CI [-0.06, 0.00], *t* = -2.26, *p* = .024); (2) for mean neuroticism scores: B = -0.01 (BCa 95% CI [-0.03, 0.01], *t* = -0.79, *p* = .429); (3) for neuroticism scores 1 *SD* above the mean: B = 0.02 (BCa 95% CI [-0.01, 0.04], *t* = 1.14, *p* = .254)

To further examine the interaction between WPC and neuroticism in mothers, a simple slope analysis was conducted using the SPSS tool PROCESS Model 1 [79], see Fig. 2. In mothers with average and high neuroticism levels, WPC was unrelated to their relationship satisfaction, so they showed consistently low relationship satisfaction. In contrast, in mothers with low neuroticism scores, WPC significantly interacted with relationship satisfaction, such that high WPC predicted lower relationship satisfaction.

## Discussion

### Discussion of the present results

The aim of this study was to investigate whether WPC at 14 months after childbirth negatively predicts parental relationship satisfaction in employed mothers and fathers around two years after the birth of their child and whether and how the Big Five personality traits interact with this association. In mothers, WPC alone did not predict relationship satisfaction, but in interaction with neuroticism, it became a significant predictor with a very small effect size. Notably, this interaction effect indicated that WPC became a significant predictor only for mothers with low neuroticism scores. For fathers, WPC alone significantly predicted relationship satisfaction with very small effect sizes, whereby higher WPC scores indicated lower relationship satisfaction. No significant interaction effects were found for the investigated personality traits in fathers. Additionally, out of the considered variables for potential confounders, the exposure to the COVID-19 pandemic and the participants’ employment status 24 months after childbirth were both not significantly linked to relationship satisfaction for either mothers or fathers and were therefore not included in further analyses. Among the confounders included in the regression analyses, higher perceived social support (small to medium effect sizes) significantly predicted higher relationship satisfaction in mothers and fathers, whereas higher academic degree was significantly related to lower relationship satisfaction only in mothers (very small effect sizes) and expecting another child at T4 was significantly related to higher relationship satisfaction only in fathers (small effect sizes).

First, it was hypothesized that higher WPC would predict lower relationship satisfaction. The present results confirmed this hypothesis only for fathers. The lack of a significant direct effect in mothers differs from the results reported in previous research: As presented in the introduction, former studies indeed found negative effects of WPC on relationship satisfaction in mothers [18] and suggested equally negative effects of WPC on relationship satisfaction among women and men [17, 83]. What could account for these differences? One theoretical explanation is sex-differential boundary management. This approach suggests that women form more robust boundaries around the family domain due to socialization and traditional values, as they still take on more care work and may feel more responsible for the wellbeing of the family and therefore increasingly protective of it [43, 84]. Accordingly, women would be less likely to experience WPC than men, which might also explain weaker effects on relationship satisfaction. Given that we also found fathers to experience significantly more WPC than mothers, this may be an accountable approach. Furthermore, our findings of a significant negative association between fathers’ WPC and their relationship satisfaction replicated previous studies [17, 85]. This finding is also supported by a corresponding theoretical approach in the literature. Previous research has proposed a “rational view” according to which there is a linear relationship between the amount of time spent in a particular domain (e.g., work and family-related tasks) and the amount of conflict experienced as a result [86]. Hence, people spending more time at work (here fathers) are at greater risk of perceiving WPC. Finally, the aforementioned approaches altogether may explain why WPC negatively predicted relationship satisfaction only for fathers in this study. In addition to these explanations, a methodological and demographic factor may also contribute to the findings: Meta-analytic evidence suggests that European countries often have more family-friendly policies and different regulations regarding working hours, which can weaken the negative association between WPC and relationship satisfaction compared to studies conducted on North American samples [17]. German regulations on maternity protection, parental leave benefits, and opportunities for part-time work may therefore represent another aspect that possibly contributes to the multifaceted array of potential explanations for the present results.

Second, it was hypothesized that every Big Five personality trait would significantly interact with WPC when predicting relationship satisfaction. However, only the interaction effect for WPC and neuroticism for mothers was found to be statistically significant, hence all other hypotheses must be rejected. With respect to neuroticism, our results align with prior research, repeatedly showing its positive association with WPC [22–24] and negative association with relationship satisfaction [25, 27, 34]. Regarding the other personality traits, embedding our results in existing research is more challenging. Although all Big Five personality traits have previously been linked to various relationship outcomes [27, 36], the investigation of the moderating role of personality on the association between WPC and relationship satisfaction has been neglected so far. Therefore, it is particularly difficult to interpret the non-significant findings. These findings could imply that the mentioned personality traits simply do not play a role in this context.

Remarkably, the hypothesized interaction effect of WPC and neuroticism in mothers was statistically significant. However, contrary to the hypothesis, it was negative. As this effect may not be intuitively comprehensible, it is explained in more detail here and then placed in the context of the present results: A negative regression coefficient of an interaction term indicates that an increase in one predictor variable weakens the significance of the effect between the other predictor variable and the outcome variable. The calculated simple slopes show how this can be applied to the present results: With increasing neuroticism, the effect of WPC on relationship satisfaction weakens. This manifested in two ways: First, when neuroticism was low, WPC negatively predicted relationship satisfaction, i.e., more WPC resulted in less relationship satisfaction. This aligns with our general assumptions and previous research as well [17, 87]. In contrast, mothers with average and high neuroticism scores showed stable relationship satisfaction and therefore no detrimental effect of WPC on relationship satisfaction. Notably, this stable level of relationship satisfaction among mothers with average and high neuroticism levels was consistently as low as the relationship satisfaction of mothers with low neuroticism and high WPC. Hence, high neuroticism levels were accompanied by consistently low relationship satisfaction, suggesting that high neuroticism is generally detrimental to relationship satisfaction and is consistent with previous research indicating a direct negative impact of neuroticism on relationship satisfaction [25, 27]. This is not surprising, given that for decades research has established neuroticism as a significant risk factor with far-reaching negative effects on various aspects of life, including several facets of physical and mental health, the quality and duration of life, as well as the perception and functioning of interpersonal relations [88, 89]. It remains surprising, however, why mothers with high neuroticism scores had stable low relationship satisfaction regardless of their perceived WPC, which was assumed to be an additional stressor threatening relationship satisfaction. One possible explanatory approach for this unexpected finding is a floor effect, indicating that mothers with high neuroticism scores are already close to the lower limit of relationship satisfaction even at low levels of WPC. Hence, the present study may underestimate the interaction effect, as mothers whose relationships deteriorated severely and eventually may have ended during the study period were not included in our analyses, as data for relationship satisfaction was missing not at random. This selection effect might have particularly affected highly neurotic individuals, who may be more prone to relationship dissolution under stress.

It is noteworthy that the confounding variable, perceived social support, exhibited small to medium positive effects on relationship satisfaction in mothers and fathers. This finding is particularly significant, as it stands in contrast to the smaller effects observed for all other predictors in the regression models. The substantial positive influence of perceived social support underscores its role as a critical protective factor for relationship satisfaction, suggesting that individuals with greater social support tend to experience higher relationship satisfaction, regardless of other challenges they may face. Moreover, it is plausible that the protective effect of social support may attenuate, or even nullify, the negative impact of WPC on relationship satisfaction. This finding highlights the importance of considering social support as a potential moderator when exploring the relationship between WPC and relationship outcomes.

### Strengths and limitations

To the best of our knowledge, this study was the first to examine the moderating influence of personality (e.g., all Big Five traits) on the association between WPC and relationship satisfaction in mothers and fathers. The families included in the study were in a sensitive developmental stage, after recently having a child, and were likely transitioning back to work after parental leave. In doing so, this study helped to address the theoretical research gaps outlined above, such as considering the impact of interpersonal differences on the association between WPC and relationship satisfaction, examining a homogeneous sample by considering only parents with young children, and complementing former WPC-related research. Additionally, it shows methodological strengths, given that we used a very large community sample, investigated effects for both mothers and fathers, included a prospective view by using measures from four different time points, and used validated instruments that are established in research. Beyond, we acknowledge that data are inevitably influenced by the context in which they are gathered. Our data collection spanned the period before, during, and after the COVID-19 pandemic, a time that impacted society on many levels. Surprisingly, exposure to the COVID-19 pandemic did not significantly correlate with relationship satisfaction and was therefore not included in the regression analyses. However, by controlling for this factor, we aim to set a standard for accounting for the broader contexts in which data are collected.

Nevertheless, there are some limitations to this study. First, moderator variables (the Big Five personality traits) were assessed before the predictor and outcome variables. While there is evidence that personality can change over time, these changes are typically small and occur over longer periods, such as several years [90, 91]. Therefore, it is plausible that our findings were, if at all, only minimally influenced by personality changes over the study period. Furthermore, only individuals who reported being in a relationship with the same person at all waves were considered. That is, individuals whose relationship satisfaction declined dramatically and ended during the study period were not included, so the investigated effects may have been underestimated. Additionally, it must be considered, that the participating mothers perceived significantly less WPC than those mothers who did not complete the study. Hence, there may be a bias underrepresenting mothers with high WPC levels. Here, the healthy worker effect may be applicable, implying that research only including employed participants is more likely to represent rather healthy individuals [66, 92]. Besides, this study used a regional sample, which may not be representative of Germany. Specifically, the percentage of participants with an academic degree in the present sample is above average [81], which is also shown in the results of the attrition analyses, indicating that participants with an academic degree rather persevered throughout the relevant measurement points of this longitudinal study. While epidemiological studies often report higher educational levels [93], it still diminishes the applicability and generalizability to the German population. Finally, the present study did not model interdependence between partners, as the analyses were specifically designed to examine intra-individual processes, which represent an important step before examining dyadic mechanisms. Consequently, no conclusions can be drawn about potential crossover effects, such as how one partner’s WPC or personality traits may relate to the other partner’s relationship satisfaction.

### Implications and future research

#### Practical implications for intervention and policy

The findings of this study hold some essential implications for mothers and fathers, likewise. We found mothers with low neuroticism levels and fathers in general to have a negative association between WPC and relationship satisfaction. In addition, perceived social support was found to have a protective effect on relationship satisfaction. This underscores the need for targeted interventions and support mechanisms aimed at addressing WPC-related challenges within the context of parenthood, enhancing relationship quality, and promoting family well-being. Especially when parents lack social support in their private environment and therefore an important protective factor for their relationship satisfaction, they need to be supported by company, state, and other institutions. Hence, work-family benefits are crucial in fostering a supportive work-family culture and its perception, which has been shown to have a mitigating effect on WPC [94]. To implement this, on the one hand, work-family benefits (e.g., flexible scheduling of working hours and appointments or on-site daycare) need to be widely accessible, and on the other hand, their utilization must be destigmatized. Destigmatization is particularly important for fathers given that previous research has found that men tend to hold more negative beliefs about flexible work policies and therefore may be less likely to make use of them [95].

Beyond structural interventions, approaches at the individual and couple level may also be beneficial. One such promising approach to protect and strengthen parental relationships may be found within the framework of positive psychology. Correia et al. [96] stated that even when stress is already avoided, it is important to actively evoke and foster positive emotions to maintain a healthy relationship. Hence, we deduce that this becomes even more crucial when external stressors add an additional burden. To better cope with this stress, it is clearly important to strengthen personal resources, as we found that higher WPC (and thus, higher stress) indeed threatened fathers’ relationship satisfaction. Interestingly, we also found this pattern in mothers with low neuroticism, who already have dispositional resources in the realm of positive psychology. This suggests that positive affectivity as a disposition is not sufficient on its own but needs to be specifically targeted in interventions. The deliberate cultivation and maintenance of positive emotions thus present a potential strategy for coping with stress. Given that mothers with high levels of neuroticism reported consistently low levels of relationship satisfaction, regardless of external stressors, it becomes even more important to implement interventions that counteract this negative dispositional influence. Such interventions using the framework of positive psychology may also be applicable here, as they have already been shown to be effective even for individuals with high levels of neuroticism [97].

#### Conceptual and methodological implications for further research

The present findings also provide several implications for future research. First on a conceptual level, it should be investigated how the interplay between WPC, personality, and relationship satisfaction unfolds when mothers experience sufficient WPC to potentially have a significant negative impact on relationship satisfaction and if this can indeed be attributed to the number of working hours, as suggested above, or additional job characteristics. Beyond that, an additional approach should also be addressed in upcoming research: Although mothers in this sample reported significantly less WPC than fathers, it is possible that the level of WPC was indeed sufficient to potentially have a negative impact on relationship satisfaction. The lack of a significant negative association between WPC and relationship satisfaction may then be attributed to unknown third variables. Therefore, it is important to explore whether there are any protective factors in this specific life phase in mothers that could mitigate the negative effect of even high levels of WPC on relationship satisfaction.

Beyond these conceptual suggestions for future research, we also provide some recommendations for the methodological implementation of future studies: First, in future investigations, a bidirectional assessment of work-family conflict is recommended to capture both dimensions of stress associated with the interplay and spillover between work and family. Moreover, future research could also expand in analyzing WPC and relationship satisfaction over the course of time. On the one hand, WPC might change in the months and years after reemployment (e.g., by adjusting working hours or childcare circumstances). On the other hand, including repeated measures may be insightful by potentially including participants whose relationship satisfaction declines to the point of relationship dissolution, in order to gain a more comprehensive understanding of the dynamics involved. Finally, this study has provided essential foundational work by examining the interaction of WPC and personality in predicting relationship satisfaction at the individual level. This was necessary to first gain a primary understanding of potential associations between these constructs. At the same time, we acknowledge that individuals are inherently embedded in social contexts and influence one another through their interactions, particularly within close relationships such as romantic partnerships, as emphasized by the Interdependence Theory [98, 99]. Building on this perspective, frameworks such as the Spillover–Crossover Model [100] suggest that experiences in the work domain not only spill over into the home domain within individuals but may also cross over to affect the well-being and relationship experiences of their partners. Consequently, future research may build on the present findings by incorporating dyadic approaches that explicitly account for these interpersonal processes by exploring whether and how personality traits and WPC interact within couples. Given that neuroticism was the only personality trait that significantly interacted with WPC when predicting relationship satisfaction in mothers, investigating its effects dyadically should be of specific interest.

## Conclusion

In conclusion, the present prospective cohort study aimed to investigate how the Big Five personality traits moderate the association between WPC and relationship satisfaction in employed parents of two-year-old children. The findings shed light on the complex interplay between these variables, offering insights into both mothers’ and fathers’ experiences. For mothers, the study revealed an unexpected pattern. While WPC alone did not significantly predict relationship satisfaction, the interaction between WPC and neuroticism played a central role. Contrary to our hypothesis, high neuroticism levels did not intensify the detrimental effect of WPC on relationship satisfaction. This detrimental effect was only shown in mothers with low neuroticism levels. Instead, mothers with average and high neuroticism levels reported consistent relationship satisfaction. However, these reported relationship satisfaction levels were rather low, indicating a floor effect. Interestingly, fathers showed a different pattern. While higher WPC was associated with lower levels of relationship satisfaction, no personality trait was a significant moderator, such that the association between WPC and relationship satisfaction remained the same regardless of the level of any of the Big Five traits. Additionally, fathers reported significantly more WPC than mothers, which may serve as an explanatory approach for the present results. Due to the various detrimental ways in which WPC and diminished relationship satisfaction can affect people’s lives, we therefore call for additional research linking WPC, relationship satisfaction, and personality to further investigate the partially unexpected findings of this study. Based on the present results we recommend the development and expansion of measures related to work-family benefits to ensure the balance between work and family responsibilities. Additionally, interventions within the realm of positive psychology offer a promising approach to enhance coping with stress in a relationship-supportive manner.

## Supplementary Information

Additional file 1.

Additional file 2.

Additional file 3.

Additional file 4.

Additional file 5.

## Data Availability

The dataset presented in this article is not publicly available because of legal and ethical constraints. Public sharing of participant data was not included in the informed consent of the study. Requests to access the datasets should be directed to the project manager and principal investigator Susan Garthus-Niegel.
