# Supplementary material for: Associations between work-privacy conflict and parental relationship satisfaction two years after childbirth: unveiling the moderating role of personality
Source: BMC Public Health. 2026 Jul 30;26:2240. doi: 10.1186/s12889-026-28783-2 (PMC13422093; doi:10.1186/s12889-026-28783-2)
Supplement: Supplementary file 1 — Additional file 1. [file 12889_2026_28783_MOESM1_ESM.docx]

**Additional file 1**

**Mean inter-item correlations for each Big Five personality trait**

**Table AF.1.1**

*Mean inter-item correlations (Pearson) for each Big Five personality trait in mothers*

| **Variable** | **Item Pair** | **Inter-item correlation** | **MIC** |
| --- | --- | --- | --- |
| **Agreeableness** | I1 ↔ I2 | 0.228** | 0.323 |
|  | I1 ↔ I3 | 0.449** |  |
|  | I2 ↔ I3 | 0.291** |  |
| **Conscientiousness** | I1 ↔ I2 | 0.361** | 0.360 |
|  | I1 ↔ I3 | 0.421** |  |
|  | I2 ↔ I3 | 0.297** |  |
| **Extraversion** | I1 ↔ I2 | 0.698** | 0.619 |
|  | I1 ↔ I3 | 0.572** |  |
|  | I2 ↔ I3 | 0.586** |  |
| **Neuroticism** | I1 ↔ I2 | 0.445** | 0.417 |
|  | I1 ↔ I3 | 0.407** |  |
|  | I2 ↔ I3 | 0.399** |  |
| **Openness to experience** | I1 ↔ I2 | 0.409** | 0.384 |
|  | I1 ↔ I3 | 0.373** |  |
|  | I2 ↔ I3 | 0.369** |  |

*Note.* MIC = Mean inter-item correlation. I1 = Item 1; I2 = Item 2; I3 = Item 3. Mean inter-item correlations are considered ideal if they lie within the range between .20 and .40, suggesting that items are both homogeneous enough but also contain unique variance according to Piedmont (74).

***p* < .01.

**Table AF.1.2**

*Mean inter-item correlations (Pearson) for each Big Five personality trait in fathers*

| **Variable** | **Item Pair** | **Inter-item correlation** | **MIC** |
| --- | --- | --- | --- |
| **Agreeableness** | I1 ↔ I2 | 0.130** | 0.238 |
|  | I1 ↔ I3 | 0.347** |  |
|  | I2 ↔ I3 | 0.238** |  |
| **Conscientiousness** | I1 ↔ I2 | 0.409** | 0.400 |
|  | I1 ↔ I3 | 0.470** |  |
|  | I2 ↔ I3 | 0.321** |  |
| **Extraversion** | I1 ↔ I2 | 0.653** | 0.570 |
|  | I1 ↔ I3 | 0.538** |  |
|  | I2 ↔ I3 | 0.520** |  |
| **Neuroticism** | I1 ↔ I2 | 0.484** | 0.428 |
|  | I1 ↔ I3 | 0.344** |  |
|  | I2 ↔ I3 | 0.457** |  |
| **Openness to experience** | I1 ↔ I2 | 0.240** | 0.291 |
|  | I1 ↔ I3 | 0.360** |  |
|  | I2 ↔ I3 | 0.272** |  |

*Note.* MIC = Mean inter-item correlation. I1 = Item 1; I2 = Item 2; I3 = Item 3. Mean inter-item correlations are considered ideal if they lie within the range between .20 and .40, suggesting that items are both homogeneous enough but also contain unique variance according to Piedmont (74).

***p* < .01.
