# Supplementary material for: Associations between work-privacy conflict and parental relationship satisfaction two years after childbirth: unveiling the moderating role of personality
Source: BMC Public Health. 2026 Jul 30;26:2240. doi: 10.1186/s12889-026-28783-2 (PMC13422093; doi:10.1186/s12889-026-28783-2)
Supplement: Supplementary file 2 — Additional file 2. [file 12889_2026_28783_MOESM2_ESM.docx]

**Additional file 2**

**Table A2.1**

*Results of t-tests and chi-square test analyses for sex differences*

|  | **Mothers** | | **Fathers** | | ***t*** | **BCa 95% CI** ^a^ | ***p*** | **Adjusted *p*** ^b^ |
| --- | --- | --- | --- | --- | --- | --- | --- | --- |
| **Variable** (measurement point) | ***n*** | ***M* ± *SD*** | ***n*** | ***M* ± *SD*** |  |  |  |  |
| *Outcome variable* |  |  |  |  |  |  |  |  |
| **Relationship satisfaction** (PFB-K; T4) | 686 | 18.3 **±** 4.6 | 702 | 18.1 ± 4.3 | 1.04 | [-0.22, 0.69] | .289 | >.999 |
| *Predictor variable* |  |  |  |  |  |  |  |  |
| **WPC** (COPSOQ; T3) | 686 | 29.1 **±** 18.1 | 702 | 33.6 ± 17.7 | -4.65 | [-6.35, -2.59] | <.001 | .007 |
| *Moderator variables* |  |  |  |  |  |  |  |  |
| **Agreeableness** (BFI-S; T2) | 685 | 16.3 **±** 2.8 | 700 | 16.1 ± 2.5 | 1.28 | [-0.11, 0.47] | .205 | >.999 |
| **Conscientiousness** (BFI-S; T2) | 684 | 16.9 **±** 2.7 | 699 | 16.1 ± 2.9 | 5.62 | [0.56, 1.13] | <.001 | .007 |
| **Extraversion** (BFI-S; T2) | 683 | 14.3 **±** 3.9 | 700 | 13.9 ± 3.9 | 1.53 | [-0.08, 0.75] | .128 | >.999 |
| **Neuroticism** (BFI-S; T2) | 685 | 11.3 **±** 3.6 | 702 | 9.2 ± 3.4 | 10.17 | [1.62, 2.41] | <.001 | .007 |
| **Openness to experience** (BFI-S; T2) | 684 | 13.7 **±** 3.6 | 702 | 14.0 ± 3.3 | -1.45 | [-0.65, 0.11] | .145 | >.999 |
| *Confounding and sociodemographic variables* |  |  |  |  |  |  |  |  |
| **Age** (T2) | 676 | 30.6 ± 3.7 | 688 | 32.9 ± 4.7 | -9.89 | [-2.70, -1.81] | <.001 | .007 |
| **Number of children** (T3) | 682 | 1.2 **±** 0.5 | 680 | 1.2 ± 0.5 | -0.93 | [-0.08, 0.03] | .358 | >.999 |
| **Relationship duration** (in days; T3) | 670 | 2981.1 **±** 1461.5 | 682 | 3011.8 ± 1435.0 | -0.39 | [-186.76, 115.30] | .706 | >.999 |
| **Social support** (FSozU; T3) | 681 | 4.3 **±** 0.6 | 692 | 4.1 ± 0.7 | 6.31 | [0.16, 0.30] | <.001 | .007 |
|  | **Mothers** | | **Fathers** | | **χ² (1)** | | ***p*** | **Adjusted *p*** ^b^ |
| **Variable** (measurement point) | ***n*** | ***%*** | ***n*** | ***%*** |  | |  |  |
| **Country of Birth** (T1) | 683 |  | 697 |  | 1.16 | | .282 | >.999 |
| Germany |  | 96.9 |  | 97.8 |  | |  |  |
| Other |  | 3.1 |  | 2.2 |  | |  |  |
| **Academic degree** (T1) | 685 |  | 687 |  | 2.58 | | .108 | >.999 |
| Yes |  | 61.9 |  | 57.6 |  | |  |  |
| No |  | 38.1 |  | 42.4 |  | |  |  |
| **COVID-19 pandemic** (T4) | 686 |  | 698 |  | 0.08 | | .776 | >.999 |
| Yes |  | 87.0 |  | 87.5 |  | |  |  |
| No |  | 13.0 |  | 12.5 |  | |  |  |
| **Employment status** (T4) | 686 |  | 702 |  | 61.99 | | <.001 | <.001 |
| Yes |  | 86.6 |  | 97.9 |  | |  |  |
| No |  | 13.4 |  | 2.1 |  | |  |  |
| **Expecting another child** (T4) | 686 |  | 702 |  | 0.10 | | .774 | >.999 |
| Yes |  | 16.5 |  | 17.1 |  | |  |  |
| No |  | 83.5 |  | 82.9 |  | |  |  |

*Note.* Two-tailed testing. BCa CI = bias-corrected and accelerated confidence interval with α = 0.05, 95% percentile, based on 2,000 bootstrap samples. T1 = during pregnancy; T2 = 8 weeks after the anticipated birth; T3 = 14 months after the actual birth date; T4 = 2 years after the actual birth date. BFI-S = Big Five Inventory-SOEP; F-SozU = German Social Support Questionnaire; WPC = Work-privacy conflict; COPSOQ = Copenhagen Psychosocial Questionnaire; PFB-K = Short Form of the Partnership Questionnaire.

^a^ Bootstrapping was only performed for *t*-tests due to lacking normal distributions in variables.

^b^ *p*-values were adjusted using Bonferroni-Holm correction.
