# Supplementary material for: Associations between work-privacy conflict and parental relationship satisfaction two years after childbirth: unveiling the moderating role of personality
Source: BMC Public Health. 2026 Jul 30;26:2240. doi: 10.1186/s12889-026-28783-2 (PMC13422093; doi:10.1186/s12889-026-28783-2)
Supplement: Supplementary file 3 — Additional file 3. [file 12889_2026_28783_MOESM3_ESM.docx]

**Additional file 3**

**Table AF.3**

*Results of chi-square test and t-tests for differences between completers and non-completers (attrition analyses)*

|  | **Completers** | | **Non-completers** | | ***t*** | **BCa 95% CI** ^a^ | ***p*** | **Adjusted *p*** ^b^ |
| --- | --- | --- | --- | --- | --- | --- | --- | --- |
| **Variable** (measurement point) | ***n*** | ***M* ± *SD*** | ***n*** | ***M* ± *SD*** |  |  |  |  |
| *Predictor variable* |  |  |  |  |  |  |  |  |
| **WPC** (COPSOQ; T3) |  |  |  |  |  |  |  |  |
| Mothers | 686 | 29.1 ± 18.1 | 67 | 37.4 ± 20.9 | 3.15 | [3.52, 13.01] | .001 | .033 |
| Fathers | 702 | 33.6 ± 17.7 | 106 | 36.2 ± 18.3 | 1.40 | [-1.15, 6.71] | .158 | >.999 |
| *Moderator variables* |  |  |  |  |  |  |  |  |
| **Agreeableness** (BFI-S; T2) |  |  |  |  |  |  |  |  |
| Mothers | 685 | 16.3 ± 2.8 | 260 | 16.2 ± 2.9 | -0.44 | [-0.52, 0.35] | .669 | >.999 |
| Fathers | 700 | 16.1 ± 2.5 | 279 | 15.7 ± 2.8 | -1.75 | [-0.76, 0.03] | .078 | >.999 |
| **Conscientiousness** (BFI-S; T2) |  |  |  |  |  |  |  |  |
| Mothers | 684 | 16.9 ± 2.7 | 258 | 16.7 ± 3.0 | -1.10 | [-0.67, 0.19] | .267 | >.999 |
| Fathers | 699 | 16.1 ± 2.9 | 279 | 15.8 ± 3.0 | -1.20 | [-0.68, 0.15] | .220 | >.999 |
| **Extraversion** (BFI-S; T2) |  |  |  |  |  |  |  |  |
| Mothers | 683 | 14.3 ± 3.9 | 259 | 14.8 ± 3.8 | 2.03 | [0.04, 1.08] | .042 | .798 |
| Fathers | 700 | 13.9 ± 3.9 | 278 | 14.4 ± 3.7 | 1.87 | [-0.04, 1.05] | .069 | >.999 |
| **Neuroticism** (BFI-S; T2) |  |  |  |  |  |  |  |  |
| Mothers | 685 | 11.3 ± 3.6 | 258 | 11.3 ± 3.7 | 0.28 | [-0.47, 0.63] | .767 | >.999 |
| Fathers | 702 | 9.2 ± 3.4 | 278 | 9.4 ± 3.4 | 0.78 | [-0.25, 0.66] | .430 | >.999 |
| **Openness to experience** (BFI-S; T2) |  |  |  |  |  |  |  |  |
| Mothers | 684 | 13.7 ± 3.6 | 259 | 14.4 ± 3.7 | 2.32 | [0.13, 1.15] | .025 | .510 |
| Fathers | 702 | 14.0 ± 3.3 | 278 | 14.0 ± 3.4 | -0.02 | [-0.45, 0.49] | .983 | >.999 |
| *Confounding and sociodemographic variables* |  |  |  |  |  |  |  |  |
| **Age** (T2) |  |  |  |  |  |  |  |  |
| Mothers | 676 | 30.6 ± 3.7 | 254 | 30.1 ± 4.0 | -1.69 | [-1.04, 0.06] | .094 | >.999 |
| Fathers | 688 | 32.9 ± 4.7 | 272 | 33.0 ± 4.8 | 0.30 | [-0.53, 0.70] | .774 | >.999 |
| **Number of children** (T3) |  |  |  |  |  |  |  |  |
| Mothers | 682 | 1.2 ± 0.5 | 81 | 1.2 ± 0.5 | 0.97 | [-0.05, 0.17] | .347 | >.999 |
| Fathers | 680 | 1.2 ± 0.5 | 126 | 1.4 ± 0.8 | 2.22 | [0.03, 0.31] | .024 | .504 |
| **Relationship duration** (in days; T3) |  |  |  |  |  |  |  |  |
| Mothers | 670 | 2981.1 ± 1461.5 | 77 | 2925.4 ± 1461.3 | -0.32 | [-370.14, 270.97] | .752 | >.999 |
| Fathers | 682 | 3011.8 ± 1435.0 | 123 | 3074.7 ± 1531.0 | 0.41 | [-251.13, 358.69] | .684 | >.999 |
| **Social support** (FSozU; T3) |  |  |  |  |  |  |  |  |
| Mothers | 681 | 4.3 ± 0.6 | 65 | 4.1 ± 0.7 | -1.70 | [-0.35, 0.01] | .086 | >.999 |
| Fathers | 692 | 4.1 ± 0.7 | 103 | 4.0 ± 0.7 | -1.28 | [-0.24, 0.03] | .201 | >.999 |
|  | **Completers** | | **Non-completers** | | **χ² (1)** | | ***p*** | **Adjusted *p*** ^b^ |
| **Variable** (measurement point) | ***n*** | ***%*** | ***n*** | ***%*** |  | |  |  |
| **Country of Birth** (T1) |  |  |  |  |  | |  |  |
| Mothers | 683 |  | 259 |  | 3.77 | | .052 | .939 |
| Germany |  | 96.9 |  | 94.2 |  | |  |  |
| Other |  | 3.1 |  | 5.8 |  | |  |  |
| Fathers | 697 |  | 279 |  | 1.64 | | .201 | >.999 |
| Germany |  | 97.8 |  | 96.4 |  | |  |  |
| Other |  | 2.2 |  | 3.6 |  | |  |  |
| **Academic degree** (T1) |  |  |  |  |  | |  |  |
| Mothers | 685 |  | 256 |  | 12.34 | | <.001 | .010 |
| Yes |  | 61.9 |  | 49.2 |  | |  |  |
| No |  | 38.1 |  | 50.8 |  | |  |  |
| Fathers | 687 |  | 278 |  | 20.13 | | <.001 | <.001 |
| Yes |  | 57.6 |  | 41.7 |  | |  |  |
| No |  | 42.4 |  | 58.3 |  | |  |  |

*Note.* Two-tailed testing. BCa CI = bias-corrected and accelerated confidence interval with α = 0.05, 95% percentile, based on 2,000 bootstrap samples. T2 = 8 weeks after the anticipated birth; T3 = 14 months after the actual birth date; BFI-S = Big Five Inventory-SOEP; F-SozU = German Social Support Questionnaire; WPC = Work-privacy conflict; COPSOQ = Copenhagen Psychosocial Questionnaire.

^a^ Bootstrapping was only performed for *t*-tests due to lacking normal distributions in variables.

^b^ *p*-values were adjusted using Bonferroni-Holm correction.
