# Supplementary material for: Associations between work-privacy conflict and parental relationship satisfaction two years after childbirth: unveiling the moderating role of personality
Source: BMC Public Health. 2026 Jul 30;26:2240. doi: 10.1186/s12889-026-28783-2 (PMC13422093; doi:10.1186/s12889-026-28783-2)
Supplement: Supplementary file 4 — Additional file 4. [file 12889_2026_28783_MOESM4_ESM.docx]

**Additional file 4**

**Full results of hierarchical regression analyses including multivariate outliers**

**Table AF.4.1**

*Multiple linear regression of mothers’ WPC, personality, and their interaction on relationship satisfaction, controlled for confounders, including multivariate outliers*

| **Variable** | ***B*** | ***SE*** | **β** | **BCA 95% CI** | ***p*** | ***R*^2^** | **Adj. *R*^2^** | ***F* for Δ*R*^2^** |
| --- | --- | --- | --- | --- | --- | --- | --- | --- |
| **Model 1** |  |  |  |  |  | .08 | .08 | 11.58*** |
| Constant | 11.47 | 1.43 |  | [8.95, 14.07] | <.001 |  |  |  |
| Academic degree | -1.02 | 0.36 | **-.11** | [-1.72, -0.28] | .007 |  |  |  |
| Number of children | -0.06 | 0.39 | -.01 | [-0.86, 0.67] | .889 |  |  |  |
| Relationship duration | 0.00 | 0.00 | -.08 | [0.00, 0.00] | .062 |  |  |  |
| Social support | 1.91 | 0.29 | **.25** | [1.41, 2.43] | <.001 |  |  |  |
| Expecting another child | 0.78 | 0.48 | .06 | [-0.07, 1.63] | .077 |  |  |  |
| **Model 2** |  |  |  |  |  | .08 | .08 | 1.06 |
| Constant | 11.60 | 1.44 |  | [9.02, 14.20] | <.001 |  |  |  |
| Academic degree | -0.94 | 0.37 | **-.10** | [-1.67, -0.17] | .013 |  |  |  |
| Number of children | -0.03 | 0.40 | -.00 | [-0.85, 0.71] | .944 |  |  |  |
| Relationship duration | 0.00 | 0.00 | -.08 | [0.00, 0.00] | .063 |  |  |  |
| Social support | 1.86 | 0.30 | **.24** | [1.34, 2.39] | <.001 |  |  |  |
| Expecting another child | 0.80 | 0.48 | .06 | [-0.05, 1.67] | .068 |  |  |  |
| WPC ^a^ | -0.01 | 0.01 | -.04 | [-0.03, 0.01] | .301 |  |  |  |
| **Model 3** |  |  |  |  |  | .11 | .10 | 3.98** |
| Constant | 12.93 | 1.49 |  | [10.23, 15.55] | <.001 |  |  |  |
| Academic degree | -0.88 | 0.37 | **-.09** | [-1.59, -0.11] | .024 |  |  |  |
| Number of children | -0.13 | 0.39 | -.01 | [-0.96, 0.60] | .743 |  |  |  |
| Relationship duration | 0.00 | 0.00 | -.07 | [0.00, 0.00] | .092 |  |  |  |
| Social support | 1.55 | 0.31 | **.20** | [1.00, 2.11] | <.001 |  |  |  |
| Expecting another child | 0.71 | 0.48 | .06 | [-0.12, 1.57] | .096 |  |  |  |
| WPC ^a^ | -0.01 | 0.01 | -.02 | [-0.03, 0.01] | .526 |  |  |  |
| Agreeableness ^a^ | 0.16 | 0.07 | **.10** | [0.04, 0.29] | .016 |  |  |  |
| Conscientiousness ^a^ | 0.08 | 0.07 | .05 | [-0.06, 0.23] | .264 |  |  |  |
| Extraversion ^a^ | -0.02 | 0.05 | -.01 | [-0.11, 0.08] | .759 |  |  |  |
| Neuroticism ^a^ | -0.14 | 0.05 | **-.11** | [-0.24, -0.03] | .013 |  |  |  |
| Openness to experience ^a^ | 0.03 | 0.05 | .02 | [-0.08, 0.13] | .621 |  |  |  |
| **Model 4** |  |  |  |  |  | .12 | .10 | 1.35 |
| Constant | 12.96 | 1.49 |  | [10.40, 15.52] | <.001 |  |  |  |
| Academic degree | -0.90 | 0.37 | **-.09** | [-1.61, -0.16] | .020 |  |  |  |
| Number of children | -0.17 | 0.39 | -.02 | [-1.00, 0.56] | .666 |  |  |  |
| Relationship duration | 0.00 | 0.00 | -.06 | [0.00, 0.00] | .125 |  |  |  |
| Social support | 1.53 | 0.31 | **.20** | [0.95, 2.11] | <.001 |  |  |  |
| Expecting another child | 0.73 | 0.48 | .06 | [-0.13, 1.65] | .097 |  |  |  |
| WPC ^a^ | -0.01 | 0.01 | -.03 | [-0.03, 0.01] | .469 |  |  |  |
| Agreeableness ^a^ | 0.17 | 0.07 | **.10** | [0.04, 0.30] | .018 |  |  |  |
| Conscientiousness ^a^ | 0.07 | 0.07 | .04 | [-0.07, 0.22] | .303 |  |  |  |
| Extraversion ^a^ | -0.01 | 0.05 | -.01 | [-0.11, 0.09] | .793 |  |  |  |
| Neuroticism ^a^ | -0.14 | 0.05 | **-.11** | [-0.24, -0.03] | .010 |  |  |  |
| Openness to experience ^a^ | 0.03 | 0.05 | .02 | [-0.08, 0.13] | .595 |  |  |  |
| WPC x Agreeableness | 0.00 | 0.00 | .01 | [-0.01, 0.01] | .721 |  |  |  |
| WPC x Conscientiousness | -0.00 | 0.00 | -.01 | [-0.01, 0.01] | .754 |  |  |  |
| WPC x Extraversion | -0.00 | 0.00 | -.02 | [-0.01, 0.00] | .583 |  |  |  |
| WPC x Neuroticism | 0.01 | 0.00 | **.09** | [0.00, 0.01] | .025 |  |  |  |
| WPC x Openness to experience | 0.00 | 0.00 | .01 | [-0.00, 0.01] | .838 |  |  |  |

*Note.* *n* = 651. WPC = Work-privacy conflict; *SE* = Standard error for unstandardized beta based on 95% bias-corrected and accelerated bootstrap confidence interval (2,000 iterations); ß = Standardized beta coefficient; Adj. *R*^2^ = Adjusted coefficient of determination. Significant standardized beta coefficients are marked in bold.

^a^ Mean-centered.

***p* < .01. ****p* < .001.

**Table AF.4.2**

*Multiple linear regression of fathers’ WPC, personality, and their interaction on relationship satisfaction, controlled for confounders, including multivariate outliers*

| **Variable** | ***B*** | ***SE*** | **β** | **BCA 95% CI** | ***p*** | ***R*^2^** | **Adj. *R*^2^** | ***F* for Δ*R*^2^** |
| --- | --- | --- | --- | --- | --- | --- | --- | --- |
| **Model 1** |  |  |  |  |  | .15 | .14 | 21.44*** |
| Constant | 11.07 | 1.13 |  | [8.63, 13.16] | <.001 |  |  |  |
| Academic degree | 0.23 | 0.33 | .03 | [-0.44, 0.90] | .501 |  |  |  |
| Number of children | -0.59 | 0.32 | -.07 | [-1.31, 0.19] | .122 |  |  |  |
| Relationship duration | 0.00 | 0.00 | -.07 | [0.00, 0.00] | .065 |  |  |  |
| Social support | 1.96 | 0.23 | **.33** | [1.52, 2.45] | <.001 |  |  |  |
| Expecting another child | 1.12 | 0.43 | **.10** | [0.33, 1.91] | .005 |  |  |  |
| **Model 2** |  |  |  |  |  | .16 | .15 | 7.44** |
| Constant | 11.21 | 1.12 |  | [8.82, 13.29] | <.001 |  |  |  |
| Academic degree | 0.33 | 0.33 | .04 | [-0.35, 1.00] | .331 |  |  |  |
| Number of children | -0.51 | 0.32 | -.06 | [-1.20, 0.25] | .178 |  |  |  |
| Relationship duration | 0.00 | 0.00 | -.06 | [0.00, 0.00] | .077 |  |  |  |
| Social support | 1.89 | 0.23 | **.32** | [1.46, 2.37] | <.001 |  |  |  |
| Expecting another child | 1.15 | 0.43 | **.10** | [0.37, 1.92] | .005 |  |  |  |
| WPC ^a^ | -0.03 | 0.01 | **-.10** | [-0.04, -0.01] | .006 |  |  |  |
| **Model 3** |  |  |  |  |  | .18 | .16 | 3.16** |
| Constant | 11.59 | 1.19 |  | [9.12, 13.82] | <.001 |  |  |  |
| Academic degree | 0.33 | 0.33 | .04 | [-0.36, 1.02] | .316 |  |  |  |
| Number of children | -0.59 | 0.32 | -.07 | [-1.28, 0.18] | .117 |  |  |  |
| Relationship duration | 0.00 | 0.00 | -.06 | [0.00, 0.00] | .073 |  |  |  |
| Social support | 1.81 | 0.25 | **.30** | [1.30, 2.40] | <.001 |  |  |  |
| Expecting another child | 1.16 | 0.43 | **.10** | [0.38, 1.92] | .003 |  |  |  |
| WPC ^a^ | -0.02 | 0.01 | **-.09** | [-0.04, -0.00] | .031 |  |  |  |
| Agreeableness ^a^ | 0.08 | 0.07 | .05 | [-0.05, 0.20] | .217 |  |  |  |
| Conscientiousness ^a^ | 0.14 | 0.06 | **.09** | [0.03, 0.25] | .009 |  |  |  |
| Extraversion ^a^ | -0.07 | 0.04 | -.06 | [-0.15, 0.02] | .149 |  |  |  |
| Neuroticism ^a^ | -0.06 | 0.05 | -.05 | [-0.16, 0.04] | .223 |  |  |  |
| Openness to experience ^a^ | 0.08 | 0.05 | .06 | [-0.03, 0.19] | .128 |  |  |  |
| **Model 4** |  |  |  |  |  | .18 | .16 | 1.06 |
| Constant | 11.75 | 1.19 |  | [9.28, 13.94] | <.001 |  |  |  |
| Academic degree | 0.37 | 0.33 | .04 | [-0.34, 1.07] | .279 |  |  |  |
| Number of children | -0.60 | 0.32 | -.07 | [-1.30, 0.17] | .106 |  |  |  |
| Relationship duration | 0.00 | 0.00 | -.07 | [0.00, 0.00] | .072 |  |  |  |
| Social support | 1.76 | 0.25 | **.29** | [1.23, 2.39] | <.001 |  |  |  |
| Expecting another child | 1.20 | 0.43 | **.11** | [0.42, 1.97] | .003 |  |  |  |
| WPC ^a^ | -0.02 | 0.01 | **-.09** | [-0.04, -0.00] | .018 |  |  |  |
| Agreeableness ^a^ | 0.10 | 0.07 | .06 | [-0.03, 0.22] | .141 |  |  |  |
| Conscientiousness ^a^ | 0.13 | 0.06 | **.09** | [0.02, 0.24] | .020 |  |  |  |
| Extraversion ^a^ | -0.06 | 0.05 | -.05 | [-0.14, 0.02] | .222 |  |  |  |
| Neuroticism ^a^ | -0.07 | 0.05 | -.05 | [-0.16, 0.02] | .189 |  |  |  |
| Openness to experience ^a^ | 0.08 | 0.05 | .06 | [-0.02, 0.19] | .129 |  |  |  |
| WPC x Agreeableness | -0.01 | 0.00 | -.06 | [-0.01, 0.00] | .147 |  |  |  |
| WPC x Conscientiousness | 0.00 | 0.00 | .02 | [-0.00, 0.01] | .567 |  |  |  |
| WPC x Extraversion | -0.00 | 0.00 | -.03 | [-0.01, 0.00] | .462 |  |  |  |
| WPC x Neuroticism | 0.00 | 0.00 | .04 | [-0.00, 0.01] | .459 |  |  |  |
| WPC x Openness to experience | -0.00 | 0.00 | -.03 | [-0.01, 0.00] | .487 |  |  |  |

*Note.* *n* = 632. WPC = Work-privacy conflict; *SE* = Standard error for unstandardized beta based on 95% bias-corrected and accelerated bootstrap confidence interval (2,000 iterations); ß = Standardized beta coefficient; Adj. *R*^2^ = Adjusted coefficient of determination. Significant standardized beta coefficients are marked in bold.

^a^ Mean-centered.

***p* < .01. ****p* < .001.
