# Supplementary material for: Associations between work-privacy conflict and parental relationship satisfaction two years after childbirth: unveiling the moderating role of personality
Source: BMC Public Health. 2026 Jul 30;26:2240. doi: 10.1186/s12889-026-28783-2 (PMC13422093; doi:10.1186/s12889-026-28783-2)
Supplement: Supplementary file 5 — Additional file 5. [file 12889_2026_28783_MOESM5_ESM.docx]

**Additional file 5**

**Full results of hierarchical regression analyses excluding multivariate outliers**

**Table AF.5.1**

*Multiple linear regression of mothers’ WPC, personality, and their interaction on relationship satisfaction, controlled for confounders, excluding multivariate outliers*

| **Variable** | ***B*** | ***SE*** | **β** | **BCA 95% CI** | ***p*** | ***R*^2^** | **Adj. *R*^2^** | ***F* for Δ*R*^2^** |
| --- | --- | --- | --- | --- | --- | --- | --- | --- |
| **Model 1** |  |  |  |  |  | .08 | .07 | 11.37*** |
| Constant | 11.35 | 1.45 |  | [8.37, 14.19] | <.001 |  |  |  |
| Academic degree | -1.00 | 0.37 | **-.11** | [-1.68, -0.30] | .006 |  |  |  |
| Number of children | 0.03 | 0.42 | .00 | [-0.80, 0.74] | .945 |  |  |  |
| Relationship duration | 0.00 | 0.00 | -.08 | [0.00, 0.00] | .051 |  |  |  |
| Social support | 1.91 | 0.29 | **.25** | [1.37, 2.49] | <.001 |  |  |  |
| Expecting another child | 0.77 | 0.48 | .06 | [-0.07, 1.63] | .070 |  |  |  |
| **Model 2** |  |  |  |  |  | .08 | .07 | 0.88 |
| Constant | 11.46 | 1.45 |  | [8.56, 14.30] | <.001 |  |  |  |
| Academic degree | -0.93 | 0.37 | **-.10** | [-1.63, -0.23] | .014 |  |  |  |
| Number of children | 0.06 | 0.42 | .01 | [-0.78, 0.76] | .895 |  |  |  |
| Relationship duration | 0.00 | 0.00 | -.08 | [0.00, 0.00] | .050 |  |  |  |
| Social support | 1.87 | 0.30 | **.24** | [1.31, 2.48] | <.001 |  |  |  |
| Expecting another child | 0.79 | 0.48 | .06 | [-0.06, 1.65] | .061 |  |  |  |
| WPC ^a^ | -0.01 | 0.01 | -.04 | [-0.03, 0.01] | .373 |  |  |  |
| **Model 3** |  |  |  |  |  | .11 | .09 | 3.76** |
| Constant | 12.79 | 1.51 |  | [9.73, 15.78] | <.001 |  |  |  |
| Academic degree | -0.88 | 0.37 | **-.09** | [-1.57, -0.19] | .020 |  |  |  |
| Number of children | -0.04 | 0.42 | -.00 | [-0.88, 0.67] | .915 |  |  |  |
| Relationship duration | 0.00 | 0.00 | -.07 | [0.00, 0.00] | .086 |  |  |  |
| Social support | 1.56 | 0.31 | **.20** | [0.98, 2.17] | <.001 |  |  |  |
| Expecting another child | 0.72 | 0.48 | .06 | [-0.12, 1.57] | .094 |  |  |  |
| WPC ^a^ | -0.01 | 0.01 | -.02 | [-0.03, 0.02] | .623 |  |  |  |
| Agreeableness ^a^ | 0.16 | 0.07 | **.09** | [0.03, 0.31] | .018 |  |  |  |
| Conscientiousness ^a^ | 0.08 | 0.07 | .05 | [-0.06, 0.23] | .260 |  |  |  |
| Extraversion ^a^ | -0.02 | 0.05 | -.01 | [-0.11, 0.08] | .729 |  |  |  |
| Neuroticism ^a^ | -0.14 | 0.05 | **-.11** | [-0.24, -0.03] | .009 |  |  |  |
| Openness to experience ^a^ | 0.02 | 0.05 | .02 | [-0.08, 0.11] | .723 |  |  |  |
| **Model 4** |  |  |  |  |  | .12 | .10 | 1.26 |
| Constant | 12.82 | 1.51 |  | [9.70, 15.75] | <.001 |  |  |  |
| Academic degree | -0.89 | 0.37 | **-.09** | [-1.58, -0.21] | .019 |  |  |  |
| Number of children | -0.09 | 0.42 | -.01 | [-0.97, 0.68] | .833 |  |  |  |
| Relationship duration | 0.00 | 0.00 | -.06 | [0.00, 0.00] | .122 |  |  |  |
| Social support | 1.54 | 0.31 | **.20** | [0.93, 2.20] | <.001 |  |  |  |
| Expecting another child | 0.74 | 0.48 | .06 | [-0.13, 1.61] | .089 |  |  |  |
| WPC ^a^ | -0.01 | 0.01 | -.03 | [-0.03, 0.01] | .536 |  |  |  |
| Agreeableness ^a^ | 0.16 | 0.07 | **.10** | [0.03, 0.31] | .019 |  |  |  |
| Conscientiousness ^a^ | 0.08 | 0.07 | .04 | [-0.07, 0.22] | .307 |  |  |  |
| Extraversion ^a^ | -0.01 | 0.05 | -.01 | [-0.11, 0.09] | .776 |  |  |  |
| Neuroticism ^a^ | -0.14 | 0.05 | **-.11** | [-0.25, -0.04] | .005 |  |  |  |
| Openness to experience ^a^ | 0.02 | 0.05 | .02 | [-0.08, 0.12] | .702 |  |  |  |
| WPC x Agreeableness | 0.00 | 0.00 | .01 | [-0.01, 0.01] | .817 |  |  |  |
| WPC x Conscientiousness | -0.00 | 0.00 | -.01 | [-0.01, 0.01] | .782 |  |  |  |
| WPC x Extraversion | -0.00 | 0.00 | -.02 | [-0.01, 0.00] | .522 |  |  |  |
| WPC x Neuroticism | 0.01 | 0.00 | **.08** | [0.00, 0.01] | .035 |  |  |  |
| WPC x Openness to experience | 0.00 | 0.00 | .00 | [-0.01, 0.01] | .972 |  |  |  |

*Note.* *n* = 647. WPC = Work-privacy conflict; *SE* = Standard error for unstandardized beta based on 95% bias-corrected and accelerated bootstrap confidence interval (2,000 iterations); ß = Standardized beta coefficient; Adj. *R*^2^ = Adjusted coefficient of determination. Significant standardized beta coefficients are marked in bold.

^a^ Mean-centered.

***p* < .01. ****p* < .001.

**Table AF.5.2**

*Multiple linear regression of fathers’ WPC, personality, and their interaction on relationship satisfaction, controlled for confounders, excluding multivariate outliers*

| **Variable** | ***B*** | ***SE*** | **β** | **BCA 95% CI** | ***p*** | ***R*^2^** | **Adj. *R*^2^** | ***F* for Δ*R*^2^** |
| --- | --- | --- | --- | --- | --- | --- | --- | --- |
| **Model 1** |  |  |  |  |  | .15 | .14 | 21.13*** |
| Constant | 11.00 | 1.14 |  | [8.91, 13.20] | <.001 |  |  |  |
| Academic degree | 0.24 | 0.33 | .03 | [-0.41, 0.88] | .466 |  |  |  |
| Number of children | -0.55 | 0.35 | -.06 | [-1.27, 0.19] | .117 |  |  |  |
| Relationship duration | 0.00 | 0.00 | -.07 | [0.00, 0.00] | .079 |  |  |  |
| Social support | 1.96 | 0.23 | **.33** | [1.51, 2.40] | <.001 |  |  |  |
| Expecting another child | 1.05 | 0.43 | **.09** | [0.30, 1.80] | .005 |  |  |  |
| **Model 2** |  |  |  |  |  | .15 | .15 | 6.71** |
| Constant | 11.15 | 1.13 |  | [9.06, 13.35] | <.001 |  |  |  |
| Academic degree | 0.33 | 0.33 | .04 | [-0.29, 0.97] | .316 |  |  |  |
| Number of children | -0.48 | 0.34 | -.05 | [-1.19, 0.27] | .177 |  |  |  |
| Relationship duration | 0.00 | 0.00 | -.06 | [0.00, 0.00] | .080 |  |  |  |
| Social support | 1.89 | 0.23 | **.32** | [1.44, 2.34] | <.001 |  |  |  |
| Expecting another child | 1.07 | 0.43 | **.09** | [0.34, 1.84] | .004 |  |  |  |
| WPC ^a^ | -0.02 | 0.01 | **-.10** | [-0.04, -0.01] | .012 |  |  |  |
| **Model 3** |  |  |  |  |  | .18 | .16 | 3.07** |
| Constant | 11.55 | 1.20 |  | [9.19, 13.88] | <.001 |  |  |  |
| Academic degree | 0.34 | 0.33 | .04 | [-0.30, 0.98] | .301 |  |  |  |
| Number of children | -0.55 | 0.34 | -.06 | [-1.27, 0.15] | .129 |  |  |  |
| Relationship duration | 0.00 | 0.00 | -.06 | [0.00, 0.00] | .082 |  |  |  |
| Social support | 1.82 | 0.25 | **.31** | [1.28, 2.36] | <.001 |  |  |  |
| Expecting another child | 1.07 | 0.43 | **.09** | [0.33, 1.82] | .004 |  |  |  |
| WPC ^a^ | -0.02 | 0.01 | **-.08** | [-0.04, 0.00] | .049 |  |  |  |
| Agreeableness ^a^ | 0.08 | 0.07 | .05 | [-0.05, 0.20] | .213 |  |  |  |
| Conscientiousness ^a^ | 0.14 | 0.06 | **.09** | [0.03, 0.25] | .022 |  |  |  |
| Extraversion ^a^ | -0.06 | 0.04 | -.06 | [-0.14, 0.02] | .149 |  |  |  |
| Neuroticism ^a^ | -0.07 | 0.05 | -.06 | [-0.16, 0.03] | .183 |  |  |  |
| Openness to experience ^a^ | 0.08 | 0.05 | .06 | [-0.03, 0.18] | .158 |  |  |  |
| **Model 4** |  |  |  |  |  | .18 | .16 | 0.97 |
| Constant | 11.71 | 1.21 |  | [9.38, 14.00] | <.001 |  |  |  |
| Academic degree | 0.37 | 0.33 | .04 | [-0.28, 1.02] | .266 |  |  |  |
| Number of children | -0.57 | 0.34 | -.06 | [-1.30, 0.18] | .115 |  |  |  |
| Relationship duration | 0.00 | 0.00 | -.06 | [0.00, 0.00] | .087 |  |  |  |
| Social support | 1.77 | 0.25 | **.30** | [1.23, 2.33] | <.001 |  |  |  |
| Expecting another child | 1.12 | 0.43 | **.10** | [0.39, 1.88] | .003 |  |  |  |
| WPC ^a^ | -0.02 | 0.01 | **-.09** | [-0.04, -0.00] | .034 |  |  |  |
| Agreeableness ^a^ | 0.10 | 0.07 | .06 | [-0.04, 0.23] | .138 |  |  |  |
| Conscientiousness ^a^ | 0.13 | 0.06 | **.08** | [0.01, 0.24] | .034 |  |  |  |
| Extraversion ^a^ | -0.06 | 0.05 | -.05 | [-0.13, 0.02] | .206 |  |  |  |
| Neuroticism ^a^ | -0.07 | 0.05 | -.06 | [-0.16, 0.01] | .147 |  |  |  |
| Openness to experience ^a^ | 0.08 | 0.05 | .06 | [-0.03, 0.17] | .153 |  |  |  |
| WPC x Agreeableness | -0.01 | 0.00 | -.06 | [-0.01, 0.00] | .123 |  |  |  |
| WPC x Conscientiousness | 0.00 | 0.00 | .02 | [-0.01, 0.01] | .658 |  |  |  |
| WPC x Extraversion | -0.00 | 0.00 | -.03 | [-0.01, 0.00] | .488 |  |  |  |
| WPC x Neuroticism | 0.00 | 0.00 | .03 | [-0.00, 0.01] | .545 |  |  |  |
| WPC x Openness to experience | -0.00 | 0.00 | -.02 | [-0.01, 0.00] | .590 |  |  |  |

*Note.* *n* = 629. WPC = Work-privacy conflict; *SE* = Standard error for unstandardized beta based on 95% bias-corrected and accelerated bootstrap confidence interval (2,000 iterations); ß = Standardized beta coefficient; Adj. *R*^2^ = Adjusted coefficient of determination. Significant standardized beta coefficients are marked in bold.

^a^ Mean-centered.

***p* < .01. ****p* < .001.
